# ROS-Induced JNK and p38 Signaling Is Required for Unpaired Cytokine Activation during *Drosophila* Regeneration

**DOI:** 10.1371/journal.pgen.1005595

**Published:** 2015-10-23

**Authors:** Paula Santabárbara-Ruiz, Mireya López-Santillán, Irene Martínez-Rodríguez, Anahí Binagui-Casas, Lídia Pérez, Marco Milán, Montserrat Corominas, Florenci Serras

**Affiliations:** 1 Departament de Genètica, Facultat de Biologia and Institut de Biomedicina de la Universitat de Barcelona (IBUB), Universitat de Barcelona, Barcelona, Spain; 2 Institute for Research in Biomedicine (IRB Barcelona), Barcelona, Spain; 3 ICREA, Catalan Institution for Research and Advanced Studies, Barcelona, Spain; The University of North Carolina at Chapel Hill, UNITED STATES

## Abstract

Upon apoptotic stimuli, epithelial cells compensate the gaps left by dead cells by activating proliferation. This has led to the proposal that dying cells signal to surrounding living cells to maintain homeostasis. Although the nature of these signals is not clear, reactive oxygen species (ROS) could act as a signaling mechanism as they can trigger pro-inflammatory responses to protect epithelia from environmental insults. Whether ROS emerge from dead cells and what is the genetic response triggered by ROS is pivotal to understand regeneration of *Drosophila* imaginal discs. We genetically induced cell death in wing imaginal discs, monitored the production of ROS and analyzed the signals required for repair. We found that cell death generates a burst of ROS that propagate to the nearby surviving cells. Propagated ROS activate p38 and induce tolerable levels of JNK. The activation of JNK and p38 results in the expression of the cytokines Unpaired (Upd), which triggers the JAK/STAT signaling pathway required for regeneration. Our findings demonstrate that this ROS/JNK/p38/Upd stress responsive module restores tissue homeostasis. This module is not only activated after cell death induction but also after physical damage and reveals one of the earliest responses for imaginal disc regeneration.

## Author Summary

Regenerative biology pursues to unveil the genetic networks triggered by tissue damage. Regeneration can occur after damage by cell death or by injury. We used the imaginal disc of *Drosophila* in which we genetically activated apoptosis or physically removed some parts and monitored the capacity to repair the damage. We found that dying cells generate a burst of reactive oxygen species (ROS) necessary to activate JNK and p38 signaling pathways in the surrounding living cells. The action of these pathways is necessary for the activation of the cytokines Unpaired (Upd). Eventually, Upd will turn on the JAK/STAT signaling pathway to induce regenerative growth. Thus, we present here a module of signals that depends on oxidative stress and that, through the p38-JNK interplay, will activate cytokine-dependent regeneration.

## Introduction

Tissues and organs need to function reliably regardless of adverse environmental conditions. Injuries, disease, infection and environmental insults are stressors causing cell damage that can be repaired via homeostatic machinery. Thus, optimal health is largely dependent upon tissue homeostasis, which involves cell replacement and tissue repair. Although many signaling pathways have been proposed to respond to environmental insults, the early activation of those signals is poorly understood.

Response to damage can involve oxidative stress and, subsequently, the stimulation of stress-activated protein kinases. The production of reactive oxygen species (ROS) by various redox metabolic reactions, which has generally been considered to be deleterious, is now emerging as an active participant in cell signaling events [1,2]. ROS are byproducts of aerobic metabolism that include superoxide O_2_^-^, peroxide H_2_O_2_ and hydroxyl radicals OH·. ROS, and in particular H_2_O_2_ are required for inflammatory cell recruitment [3,4]. Amphibian and zebrafish injuries produce the ROS necessary to promote proliferation and regeneration [5–8]. In mammalian cells, ROS are known to act as second messengers to activate diverse redox-sensitive signaling transduction cascades, including the stress-activated MAP kinases p38 and the Jun-N Terminal kinase (JNK) [9–11]. ROS-mediated p38 activation occurs during the inflammatory response in rats [12] and during the loss of self-renewal and differentiation in glioma-initiating cells [13]. It has also been found that p38 and JNK are differentially required during repair. In endothelial cells, TNF-α stimulates repair through the positive action of JNK and negative regulation of p38 [14], whereas in corneal repair, p38, and not JNK, is required for epithelial migration [15]. In *Drosophila* both MAPK have been associated with stress responses [16]. *Drosophila* p38 pathway responds to different environmental stimuli and stressors [17,18]. Moreover, increasing ROS beyond basal level triggers precocious differentiation of *Drosophila* hematopoietic progenitors through JNK signaling [19].

The JNK signaling pathway has emerged as an early response to cell death and physical damage and appears to play a critical role in compensatory proliferation, regeneration and wound healing [20–28]. Moreover, upon apoptotic stimulus p53 and JNK are activated by the caspase Dronc and function upstream of pro-apoptotic genes, creating an amplifying loop that ensures cell death [29–33]. One of the early known responses to cell death is the transcriptional activation of the phosphatase *puckered* (*puc*), a downstream effector of the JNK pathway and a powerful negative regulator of the same pathway. Interestingly *puc* has been found in surviving cells of nearby tissue after cell death [23,27] and after physical injury [22,34]. JNK activation of the cytokines *unpaired* (*upd*), a family of cytokines linked to the human interleukin-6, is necessary for hyperproliferation in *Drosophila* tumors and for wound healing [34–36]. Thus, we hypothesize here that the activation of JNK, which is amplified in dying cells, is in some way propagated to nearby surviving tissue where beneficial low levels of JNK promote *upd* expression.

Apoptotic cells have been observed in the early regeneration of different animals and are thought to provide signals that regulate wound healing and regeneration [37–39]. As apoptosis has been associated with oxidative stress and cytokines act as a functional link between oxidative stress and compensatory proliferation in mammals [40], we decided to investigate whether ROS occur upstream from the stress-activated protein kinases p38 and JNK and cytokines during tissue repair. We took advantage of the regeneration capacity of *Drosophila* imaginal disc epithelium (reviewed in [41,42]) to address these questions. Imaginal discs are larval epithelial sacs that possess a robust ability for homeostatic cell renewal to overcome the effect of stressors. We report here that, either by inflicting a physical lesion or after inducing cell death, imaginal disc cells produce ROS that are linked to the activation of p38 and JNK stress MAP kinases. In addition, JNK and p38 activity in the living tissue triggers transcription of the cytokine *unpaired* (*upd*), which acts as a ligand of the JAK/STAT signaling pathway and promotes regeneration of the missing part.

## Results

### ROS are produced after tissue damage

**Fig 1:**
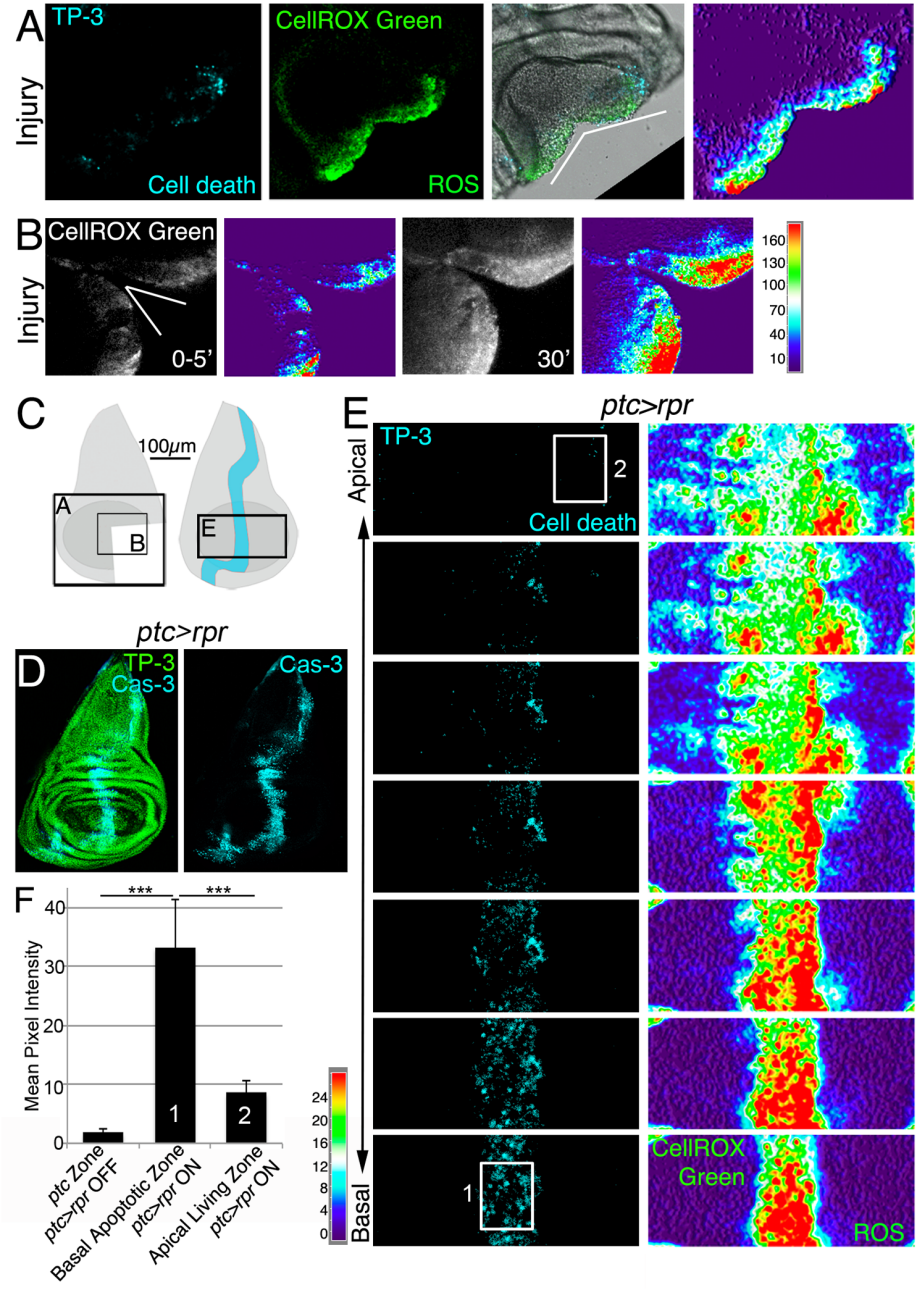
ROS produced after physical injury and after cell death. (A) Cut disc cultured ex vivo (white wedge indicates cut edges) and thermal LUT of CellROX Green. (B) Cut disc cultured ex vivo, imaged at just after cut (0–5’) and 30’ later. Thermal scale indicates pixel intensity. (C) Sketch of wing imaginal discs with the area (black square) shown in A, B and E. (D) Fixed disc stained for nuclei to show disc contour (TP-3: TO-PRO-3) and caspase-3 after *ptc>rpr* activation for 11h at 29°C. (E) *ptc>rpr* disc cultured ex vivo; basal images at the bottom, apical at the top. Left, cell death (TO-PRO-3). Right, thermal LUT taken from the ROS channel (CellROX Green) of the same preparation. Note that most dead cells (TO-PRO-3 positive) show high ROS (red in thermal image) whereas living cells (TO-PRO-3 negative) had low ROS (green-cyan in thermal image). (F) Mean pixel intensity (grey value) of the indicated zones in control discs without cell death (*ptc>rpr* OFF) and discs with cell death (*ptc>rpr* ON). The pixel intensity in the *ptc* domain in the absence of cell death (*ptc>rpr* OFF) was 1.76 ± 0.55 (SD; from 48 regions of interest [ROI] in n = 5 discs). The mean pixel intensity for the apoptotic region (basal; *ptc>rpr* ON) was 33.14±8.18 (SD), measured in 27 ROI on confocal images taken from n = 6 discs. Living cells adjacent to the apoptotic zone showed a mean grey value of 8.51±2.12 (SD; 15 ROI from 6 discs taken from cells near the *ptc* domain). White rectangles in E: example ROI for Basal Apoptotic Zone (1) and Apical Living Zone (2). The ROI’s for the *ptc* Zone, in discs in which *ptc>rpr* is OFF, were placed as (1). ***P<0.001. Thermal scale indicates sample values from raw images.

To monitor ROS after tissue damage we used CellROX Green, a cell-permeant fluorogenic probe that is non-fluorescent in the reduced state and exhibits bright fluorescence upon oxidation. We found high levels of CellROX Green near the wound edges of physically cut wing imaginal discs. Only a few of the CellROX Green positive cells were TO-PRO-3 positive cells (dying cells), indicating that most ROS-producing cells were alive (Fig 1A). We examined the production and propagation of ROS over time. Few minutes after cut (0–5’) some cells at the wound edges were CellROX Green positive, indicating that the oxidative burst is rapidly occurring after damage (Figs 1B and 1C and S1). Ex vivo imaging showed that fluorescence propagates to the neighboring cells during the first 30’ after damage (Figs 1B and S1).

We next monitored ROS production after controlled induction of cell death (also known as genetic ablation), which can be used as a type of insult to study cellular responses. Apoptosis was induced using *patched* (*ptc*)-*Gal4* to drive expression of the pro-apoptotic gene *reaper* (*rpr*) under the control of a UAS (henceforth *ptc>rpr*); the Gal4/UAS system was controlled by the temperature-sensitive form of Gal80 (*Gal80* ^*TS*^), which inhibits Gal4 and enables examination of regeneration after cell death [23,24]. As previously described, *ptc>rpr* discs show a stripe of apoptotic cells that eventually extrudes basally and is replaced apically by living cells (Fig 1D) [23]. CellROX Green was strongly incorporated into the *ptc>rpr* apoptotic cells (TO-PRO-3 positive) (Fig 1E). Strikingly, living cells adjacent to the apoptotic zone also showed ROS, albeit at much lower levels than in dead cells (Fig 1E and 1F). Similar observations were obtained using 2',7'-dichlorodihydrofluorescein diacetate (H2DCFDA) which upon oxidation is converted to the highly fluorescent DCF. Indeed, cut or *rpr*-ablated discs, showed high levels of fluorescence on the wound edges, in the apoptotic cells and also in the living cells near the apoptotic (S1B, S1C and S1D Fig).

Thus, these results showed that both physical injury and genetically induced apoptosis are insults that result in the production of ROS.

### ROS are required for tissue repair

Oxidative burst following death or damage could propagate from dying to living cells in sub-toxic doses and initiate repair. To explore this issue, we decided to deplete ROS production and examine adult wings after cell death. We first checked whether antioxidants (vitamin C, Trolox or N-acetyl cysteine [NAC]) are capable of blocking ROS production. We incubated cut discs in Schneider’s medium containing antioxidants, and found strong reduction of CellROX Green fluorescence (S2 Fig).

**Fig 2:**
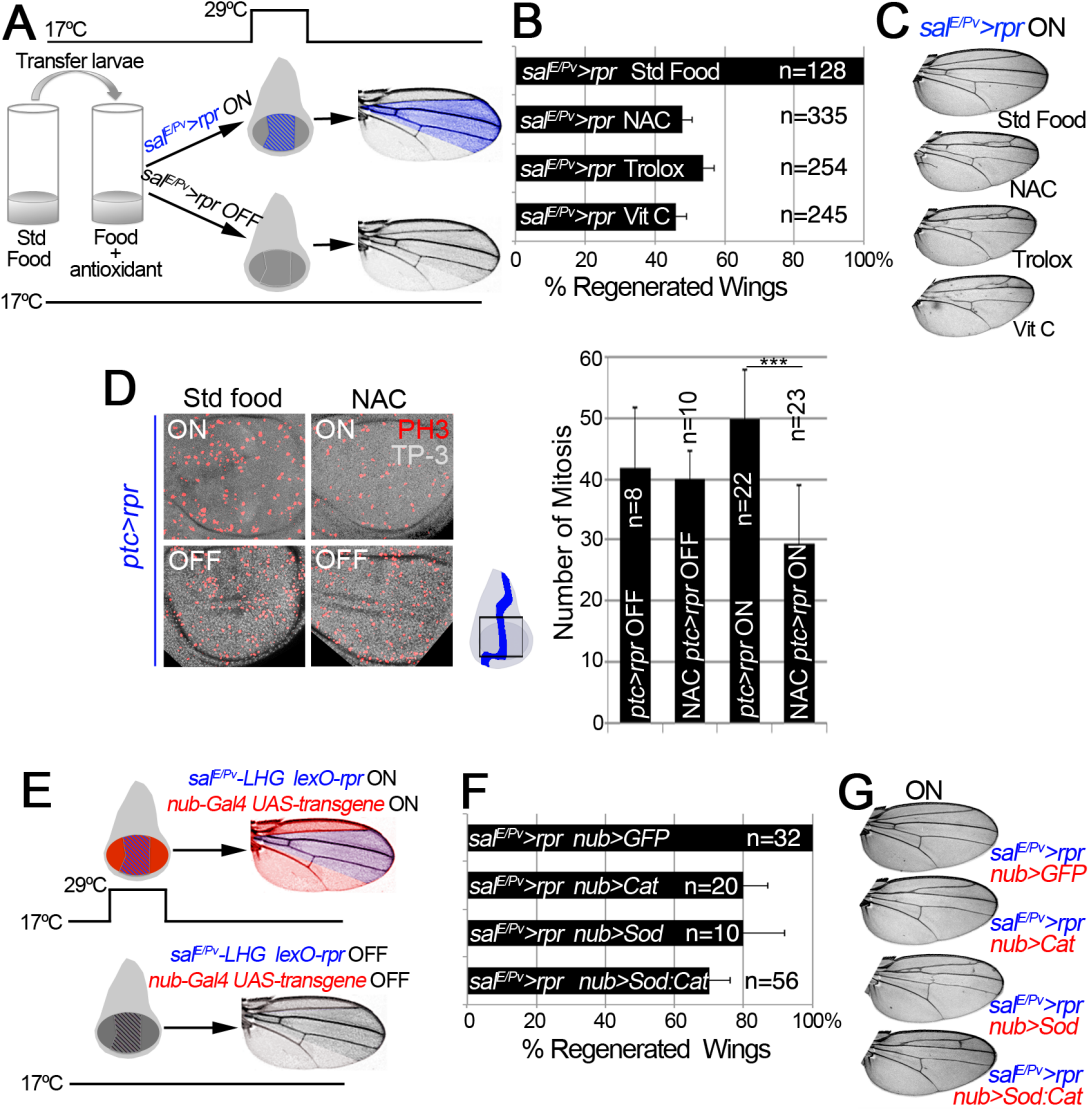
ROS are required for tissue repair. (A) Design for chemical antioxidant intake and cell death induction. At 17°C and 24 h before cell death induction, larvae were transferred to a vial with standard food supplemented with antioxidant. Cell death (*sal* ^*E/Pv*^ *>rpr* ON) was induced by shifting temperature to 29°C for 11 h (blue stripes in the disc). Larvae were transferred to 17°C where they regenerated and emerged into adults, in which wings were scored. Blue color in the wing: area emerged from *sal* ^*E/Pv*^. Controls *sal* ^*E/Pv*^ *>rpr* OFF were kept at 17°C to avoid cell death. (B) Percentage of regenerated wings after cell death (*sal* ^*E/Pv*^ *>rpr* ON) in the absence of antioxidant (Std Food), or in the presence of antioxidants (NAC, Trolox or Vitamin C). (C) Examples of *sal* ^*E/Pv*^ *>rpr* ON wings with the indicated food supplement. In controls without antioxidants (Std Food), the complete wing recovered. For each antioxidant an example of incomplete regeneration after cell death induction is shown. (D) Mitosis number in *ptc>rpr* discs from larvae fed with and without NAC and with or without *rpr*-ablation (ON versus OFF). *Ptc>rpr* OFF: 41.86 ± 9.84 (S.D.); NAC *ptc>rpr* OFF: 39.9 ± 4.68 (S.D.); *ptc>rpr* ON: 49.73 ± 8.18 (S.D.); NAC *ptc>rpr* ON: 29.52 ± 9.41 (S.D.) (E) Design for ectopic expression of enzymatic antioxidant transgenes and simultaneous cell death induction when shifted to 29°C for 11 h. The Gal4/UAS (red) activate Cat, Sod or Cat+Sod transgenes. Blue striped area: *sal* ^*E/Pv*^ *-LHG lexO-rpr*. Adult wings were scored for complete regeneration of the missing zone. Red coloration indicates zone influenced by the enzymatic antioxidant; purple: zone influenced by enzymatic antioxidant and cell death. *sal* ^*E/Pv*^ *-LHG* and *nub-Gal4* are under the control of *tubGal80* ^*TS*^. (F) Percentage of regenerated wings in Cat, Sod or Cat and Sod ectopically expressed transgenes. (G) Wings from individuals after cell death and transgene activation (ON). For Cat, Sod or Sod:Cat and example of incomplete regeneration is shown. TP-3: TO-PRO-3. ***P<0.001

Next, we studied the effects of ROS scavenging on regeneration. We used a Gal4 construct under the control of a wing-specific enhancer (*sal* ^*E/Pv*^ *-Gal4*), which allows analysis in adult wings while not affecting the rest of the organism, to activate *UAS-rpr* (henceforth *sal* ^*E/Pv*^ *>rpr*). To deplete intracellular ROS, *sal* ^*E/Pv*^ *>rpr* larvae were fed with food supplemented with antioxidants (Fig 2A). ROS scavengers in *sal* ^*E/Pv*^ *>rpr* controls kept at 17°C to prevent cell death did not show any alteration of wing morphology (S2B Fig). Conversely, a *sal* ^*E/Pv*^ *>rpr* control group without scavengers moved to 29°C for 11 h showed complete wing regeneration (Fig 2B). However, the *sal* ^*E/Pv*^ *>rpr* experimental group with ROS scavengers and induced cell death showed incomplete regeneration in about 50% of the cases (Fig 2B and 2C). We considered incomplete regeneration when some veins or intervein sectors were missing. To discard that these effects could be caused by differences in survival or developmental delay, we checked whether proliferation is impaired after ROS depletion. We counted the number of mitoses after cell death induction in discs from NAC-fed larvae and found a significant decrease compared to discs from larvae fed in the absence of antioxidants (Fig 2D). The number of mitoses in controls fed with or without antioxidants and kept at 17°C to block cell death did not vary (Fig 2D).

In addition, we used enzymatic manipulation of ROS. Superoxide dismutase (Sod) catalyzes the dismutation of superoxide anion into oxygen and hydrogen peroxide. In the presence of hydrogen peroxide, Catalase (Cat) catalyzes its breakdown into water and oxygen. Thus, overexpression of Sod or Cat will remove their respective ROS substrates, whereas simultaneous activation of Sod and Cat will enhance the depletion of both O_2_^-^ and H_2_O_2_. *UAS-Sod*, *UAS-Cat* or *UAS-Sod*:*UAS-Cat* were ectopically expressed under the *nub-Gal4* driver, which operates throughout the wing pouch (Fig 2E). To induce cell death, we used an independent transactivator based on the LexA/lexO binary system. We generated a *sal* ^*E/Pv*^ *-LHG* transgene, which includes a Gal80 suppressible form of LexA [43], to conditionally express *lexO-rpr* in the *sal* ^*E/Pv*^ domain. This combination permits control of the temporary expression of two binary systems (*sal* ^*E/Pv*^ *-LHG lexO-rpr* and *nub-Gal4 UAS-*transgene) by *tubGal80* ^*TS*^ (Fig 2E). This design has the advantage of simultaneously activating two wing-specific transgenes (*nub-Gal4* and *sal* ^*E/Pv*^ *-LHG*) in overlapping domains, therefore hindering early ROS. After *sal* ^*E/Pv*^ *-LHG lexO-rpr* genetic ablation and *nub-Gal4 UAS-*transgene expression we allowed the larvae to develop to adulthood and found a drop in the number of regenerated wings (Figs 2F and 2G and S2C and S2D). Together, these results indicate that chemical and enzymatic ROS scavengers interfere with regeneration.

### ROS control JNK activity

**Fig 3:**
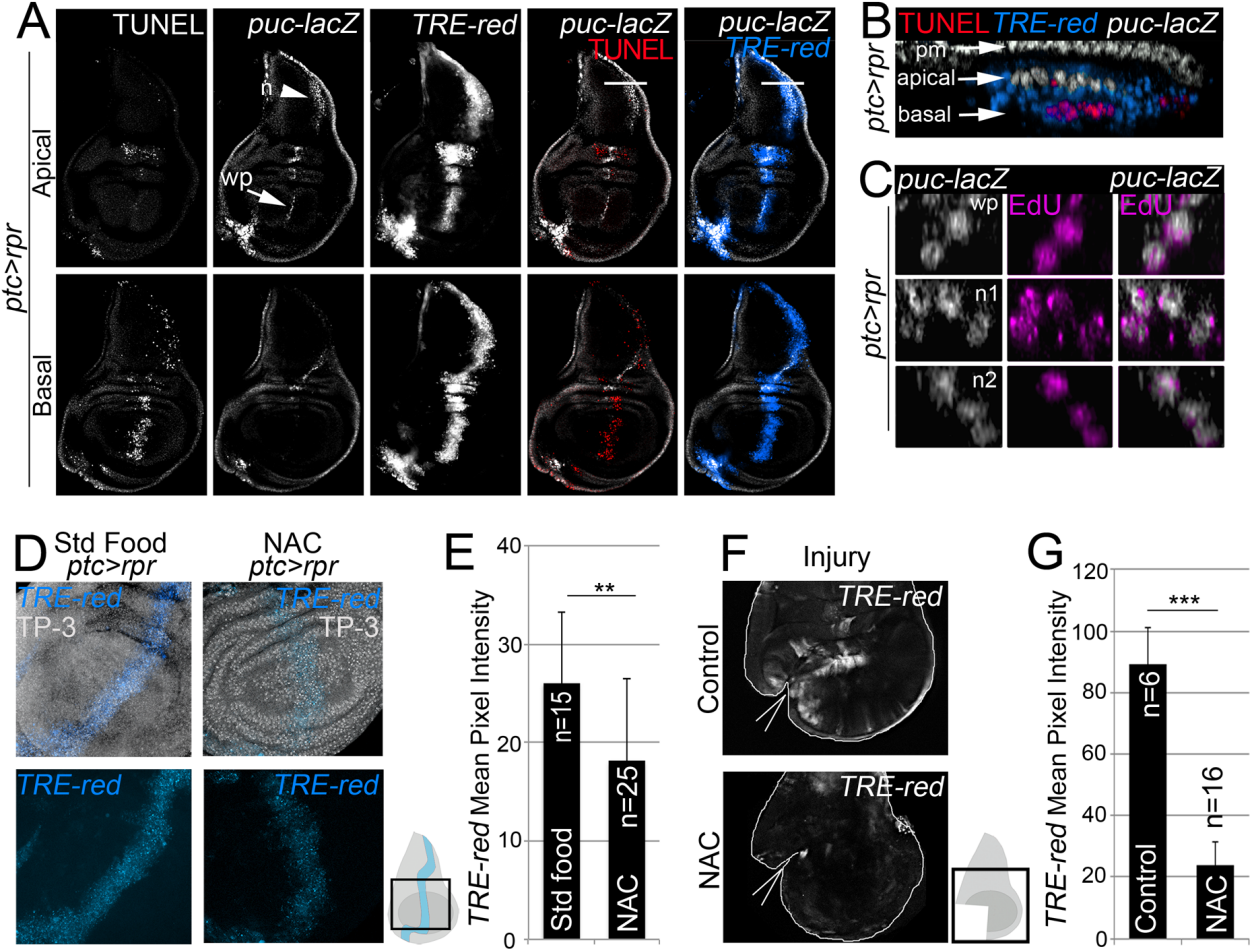
ROS control JNK activity. (A) Test of JNK reporters. All images in A correspond to the same disc after *ptc>rpr* induction. Top row: apical sections. Bottom row: basal sections. Note that *puc* is more abundant in apical than basal sections, particularly in the notum (n; arrowhead) and wing pouch (wp; arrow). Cell death (TUNEL) and high *TRE-red* are more abundant in basal sections. (B) Zoom of a digital cross section of the zone marked with a white line in A. Endogenous *puc-lacZ* is found in the outer layer of peripodial membrane cells (pm). *Puc-lacZ* cells in the disc columnar epithelium are apical (white), most apoptotic cells are basal (red), and *TRE-red* positive cells are apical and basal (blue). (C) Three digital cross section in an apical *puc-lacZ* zone of the wing pouch (wp) and notum (n1, n2). Each example contains three to four cells with co-localization of ß-galactosidase and EdU. (D) *TRE-red* reporter in *ptc>rpr* discs of larvae fed with standard food or NAC-supplemented food (NAC). TP-3: TO-PRO-3. (E) Mean pixel intensities of *TRE-red* reporter in *ptc>rpr* discs with standard or NAC food. The pixel intensity for standard food was 26.06 ± 7.22 (S.D.; n = 15) and for NAC 18.12 ± 8.32 (S.D.; n = 25). (F) *TRE-red* reporter expression in physically injured discs, cultured for 7 h ex vivo in Schneider’s culture medium with or without NAC. Outline: disc contour. Wedges: cut. (G) Mean pixel intensities of *TRE-red* reporter in ex vivo cultured discs with or without NAC. The pixel intensity for standard culture was 88.98 ± 22.25 (S.D.; n = 6) and for NAC 23.98 ± 10.26 (S.D.; n = 16). **P<0.01, ***P<0.001.

To determine whether ROS act on JNK during wing disc repair, we first monitored the activity of this pathway in wing discs after cell death. We used two different reporters to monitor JNK activity: *puc-lacZ*, which marks *puc*-expressing cells [44], and the *TRE-DsRed*.*T4* reporter, which monitors the JNK substrate *AP1* transcription factor (hereafter *TRE-red* reporter) (Fig 3A and S3A Fig) [45]. In *ptc>rpr* discs, we found high levels of *TRE-red* reporter in the basal apoptotic zone and, to a lesser extent, in the apical living cells (Fig 3A and 3B). In contrast, *puc-lacZ* positive cells were found in the apical zone, as described previously [23], and rarely in the apoptotic zone. Some *puc* positive cells incorporated EdU, supporting that JNK is also induced in living cells (Fig 3C).

As NAC is an excellent source of sulfhydryl SH- groups and efficiently promotes scavenging of free radicals [46], it was the most suitable antioxidant to determine the relationship between ROS and JNK in stressed imaginal discs. To test NAC effects on JNK, we used the *TRE-red* reporter because is more rapidly and extensively expressed than *puc-lacZ* (Figs 3A and S3B) and because its activity is blocked in JNK mutants [45] or after chemical JNK inhibitors (S3C Fig). We found that the mean pixel intensity of the *TRE-red* reporter in *ptc>rpr* wing discs from animals grown in NAC-supplemented food was lower than in the same zone of individuals fed with standard food (Fig 3D and 3E). Moreover, discs cultured ex vivo in which NAC was added into the medium resulted in a drop of *TRE-red* activity after physical injury (Fig 3F and 3G). These observations indicate that activation of JNK is ROS dependent.

### ROS stimulates p38 phosphorylation

**Fig 4:**
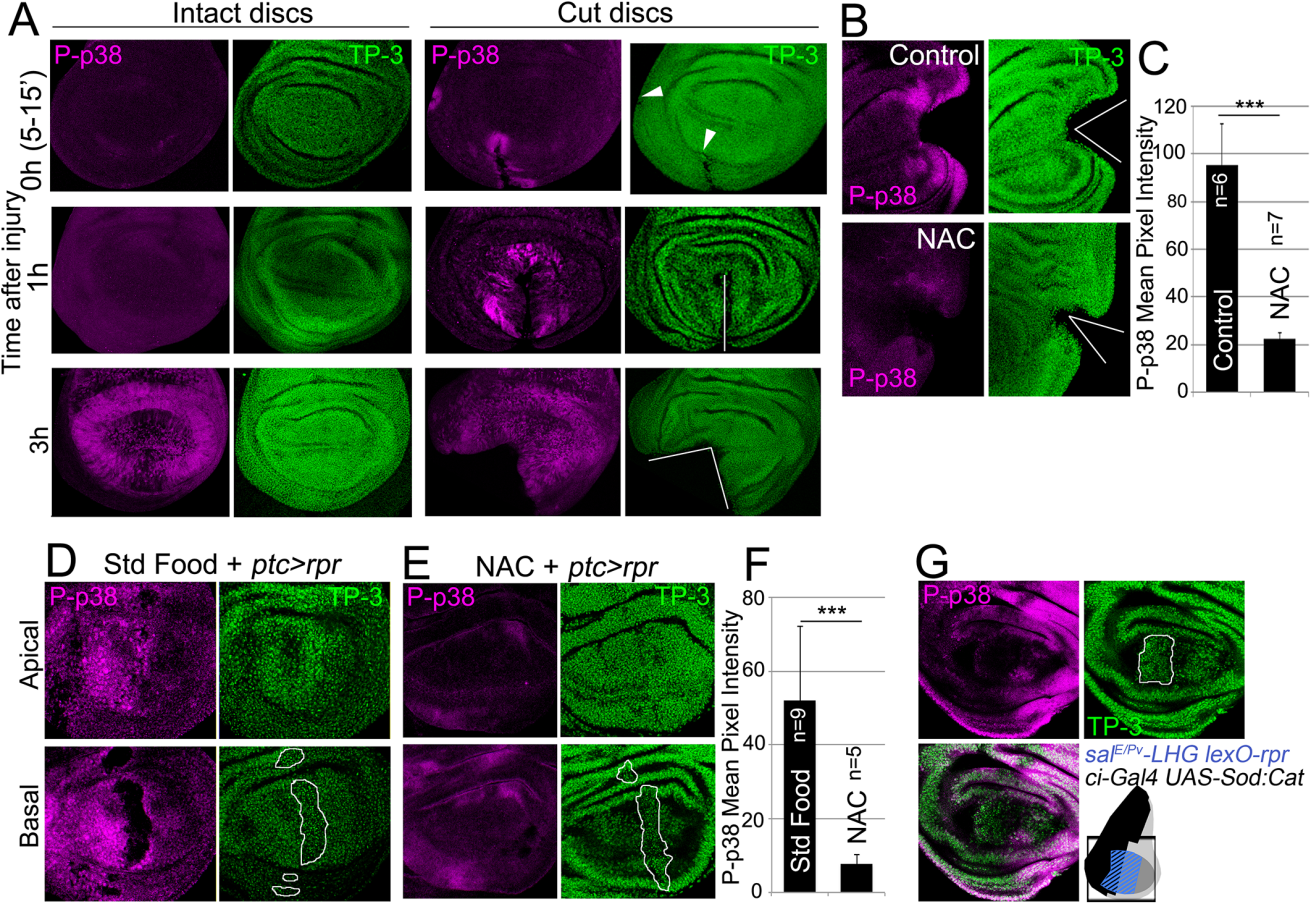
ROS stimulate p38 phosphorylation. (A) P-p38 staining of intact (uncut, controls) and cut discs cultured for the indicated times after injury. White lines: wound edges; white arrowhead: small incision. (B) Discs cultured with or without NAC, cut and stained for P-p38. (C) Mean pixel intensities of P-p38 fluorescence from cut discs cultured with standard medium (95.29 ± 17.52; S.D.) or NAC-supplemented (22.45±2.56; S.D.). (D) Apical and basal images of P-p38 after *ptc>rpr* induction. (E) Apical and basal images of *ptc>rpr* after NAC supplementation showing reduction of P-p38 localization. (F) Mean pixel intensities of P-p38 fluorescent labeling from *ptc>rpr* discs fed with standard (52.17±19.96; S.D.) or NAC-supplemented food (7,85 ± 2,42; S.D.). (G) Genetic ROS scavenging using *ci>Sod*:*Cat*, activated in the anterior compartment (*ci*, black in the sketch). *Sal* ^*EPv*^ *>rpr* cell death (blue in the sketch) in the same disc results in inhibition of P-p38 only in anterior compartment. TP-3: TO-PRO-3 nuclei staining. Outlined white in D, E and G: apoptotic zone. ***P<0.001.

Another potential response to ROS increase is the activation of the p38 signaling cascade [10,17,18]. Active p-38 signaling can be monitored using anti-phosphorylated p38 (P-p38). We found that discs fixed and incubated with anti-P-p38 a few minutes after physical injury showed P-p38 staining around the wound. P-p38 localization was variable and depended on the severity of the injury. In contrast, intact discs immediately stained after fixing did not show P-p38 (Fig 4A). However, discs cultured for 3 to 8 hours with or without injury showed P-p38 staining throughout the disc. This general staining is likely due to the stress generated by culturing, and contrasts with the fast local P-p38 response around the damaged zone. We next wondered whether the boost in ROS that propagates to the surviving tissue triggers p38 activation. We observed that the early P-p38 staining was blocked in discs cut and cultured ex vivo in medium containing NAC (Fig 4B and 4C).

We also analyzed p38 activation after inducing cell death and found P-p38 only in living cells but never in the basal apoptotic zone (Fig 4D). In the absence of cell death, no P-p38 was detected. Blocking of ROS production with NAC resulted in a significant drop in P-p38-labeled cells (Fig 4D and 4E). In addition, we used the double transcriptional trans-activator system consisting of the *sal* ^*E/Pv*^ *-LHG lexO-rpr* to induce apoptosis and simultaneously interfere with ROS production by inducing *UAS-Sod*:*UAS-Cat* in the anterior (*ci-Gal4*) compartment (Fig 4G). The results showed a strong reduction of P-p38 in the anterior (*ci-Gal4 UAS-Sod*:*UAS-Cat*) compartment in comparison to the posterior.

To test whether an independent source of ROS could activate P-p38 in discs, we fed larvae for 2h with food supplemented with 1% H_2_O_2_ and checked for P-p38. Intact discs (no cut, no cell death) from these larvae resulted in high levels of P-p38 as well as high CellROX Green fluorescence (S4 Fig).

Together, these observations show that chemical (NAC) or genetic (*UAS-Sod*:*UAS-Cat)* ROS scavengers inhibit P-p38 and therefore indicate that oxidative stress is required for p38 activation.

### p38 signaling is required for tissue repair

**Fig 5:**
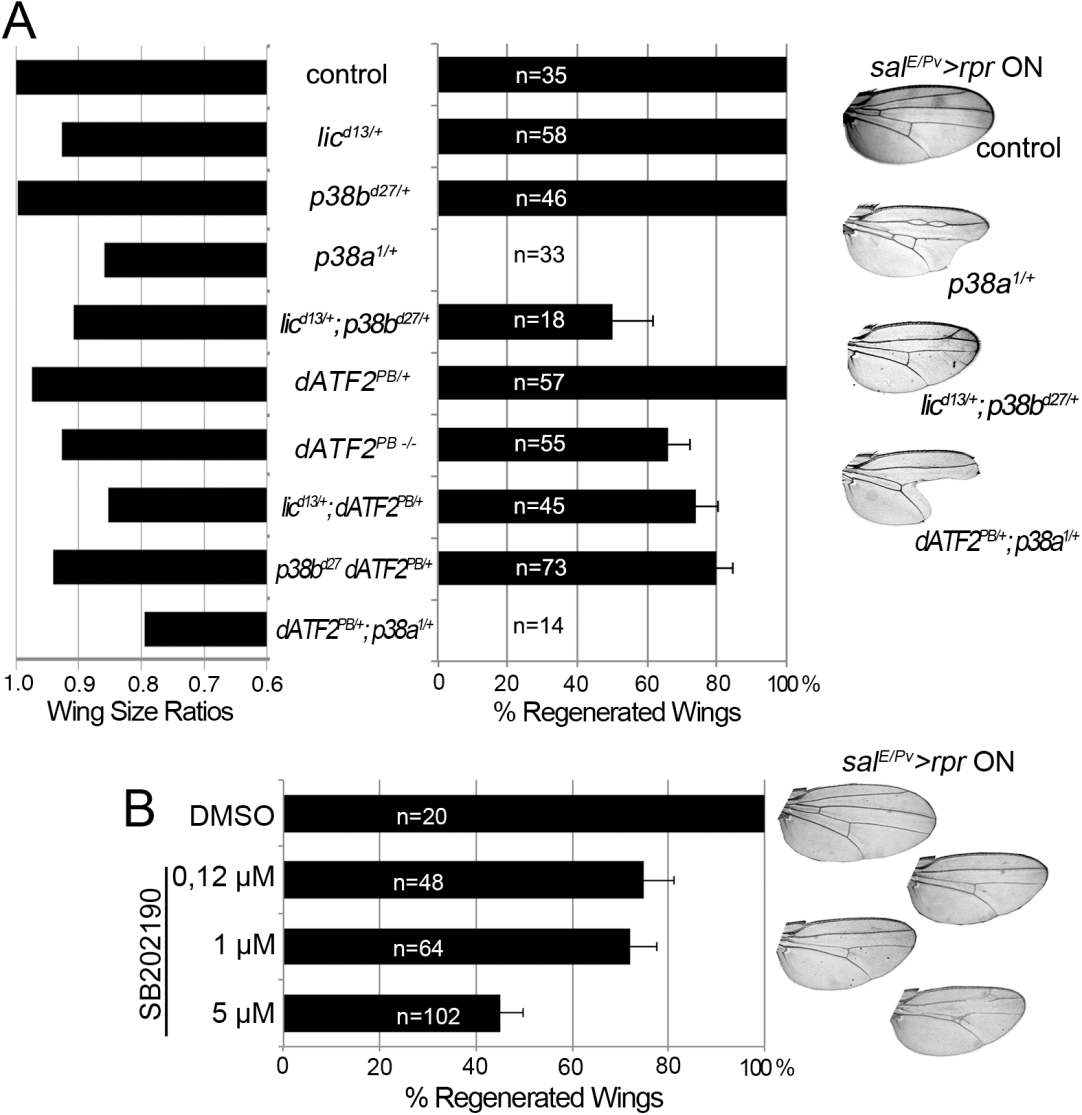
p38 inhibition impairs tissue repair. (A) Adult wing parameters in p38 signaling mutant backgrounds after genetic ablation. Left: ratios of the wing areas between experimental groups (*rpr* induction *sal* ^*E/Pv*^ *>rpr* ON) and control (no *rpr* induction *sal* ^*E/Pv*^ *>rpr* OFF). Right: percentage of fully regenerated wings. Far right: examples of wings with full regeneration (control) and incomplete regeneration (indicated genotypes) after *sal* ^*E/Pv*^ *>rpr*. (B) Percentage of fully regenerated wings after SB202190 intake in *sal* ^*E/Pv*^ *>rpr* flies. Right: wing fully regenerated (top) and examples of incomplete regeneration for each SB202190 concentration.

We next scored wing regeneration after *sal* ^*E/Pv*^ *>rpr* induction of cell death in different mutant backgrounds of the p38 pathway. As most of the alleles are lethal or semilethal in homozygosis [47], we tested them in heterozygosis. Alleles of two *Drosophila* p38 genes, *p38a* and *p38b*, were used in this work. We found that heterozygous *p38b* ^*d27*^ animals regenerated entire wings (Fig 5A). However, a severe effect was observed with *p38a* ^*1*^ as the resulting wings lacked some sectors and presented notches in the margin. *Drosophila* p38 signaling is activated by MKK3/licorne (*lic*)-mediated phosphorylation [48]. Heterozygous *lic* ^*d13*^ showed all wing sectors albeit wings were smaller than controls. However, double heterozygotes for *lic* ^*d13*^ and *p38b* ^*d27*^ were unable to regenerate some wing sectors. We also tested *Atf2* ^*PB*^, a hypomorphic allele of the ATF2 transcription factor downstream of p38 [49], either in homozygosis or in double heterozygous combinations (*lic* ^*d13*^ *Atf2* ^*PB*^ or *p38b* ^*d27*^ *Atf2* ^*PB*^). We found defects in size and pattern after *sal* ^*E/Pv*^ *>rpr* induction. Regeneration was severely impaired in double heterozygotes for *p38a* ^*1*^ (MAPK) and *Atf2* ^*PB*^ (Fig 5A).

We also blocked the pathway with UAS-RNAi constructs for *lic*, *p38b*, *p38a* and *Atf2* and analyzed the adult wings. These transgenes were activated in the anterior compartment (ci>RNAi) and cell death was induced in the *sal* ^*E/Pv*^ domain (*sal* ^*E/Pv*^ *-LHG lexO-rpr)*. We found a reduction of individuals capable to fully regenerate wings for those RNAi’s (S5A Fig).

To gain further insight into the requirement for p38, we chemically blocked the pathway using the imidazole drug SB202190, a specific cell permeable p38 MAP kinase inhibitor that has been reported to do not interfere JNK or ERK kinases and is known to prevent phosphorylation of Atf2 in *Drosophila* S2 cells [50]. We first tested the specificity of the SB202190 on P-p38 in *rpr*-ablated discs and found significant differences between individuals fed with the drug and controls. In contrast, the differences on *TRE-red* reporter were not significant (S5C Fig). This indicates that SB202190 strongly blocked P-p38 and weakly the *TRE-red*. *Sal* ^*E/Pv*^ *>rpr* larvae grown at 17°C to prevent cell death and fed with food containing SB202190 (0.12, 1.0 or 5.0 μM) emerged into normal adults (S5B Fig). However, *sal* ^*E/Pv*^ *>rpr-*induced larvae fed with SB202190 developed wings lacking some sectors. The highest percentage of aberrant wings was found using 5 μM SB202190 (Fig 5B). This observation confirms that activation of p38 is required for wing repair.

### p38 and JNK act independently

**Fig 6:**
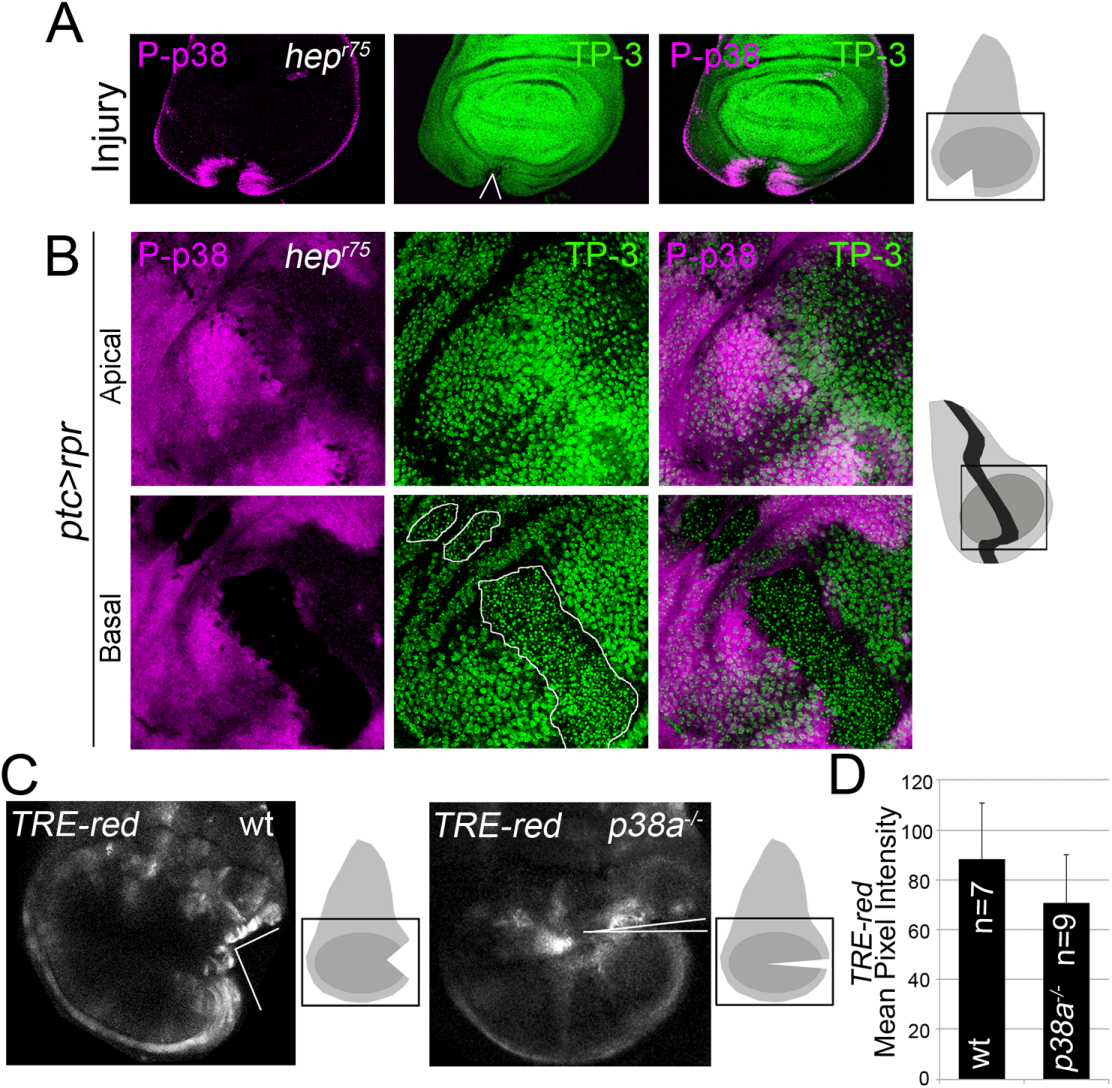
p38 and JNK are activated independently. (A) *Hep* ^*r75*^ hemizygous disc cut (wedge) and stained with P-p38. Sketch of wing discs with square indicate location of images. (B) *Hep* ^*r75*^ hemizygous disc after *ptc>rpr* induction and stained for P-p38. Dead domain is outlined white. TP-3; TO-PRO-3. (C) Wild type and *p38a* ^*1-/-*^ discs, cultured for 7h showing *TRE-red* activation close to the cut edges. (D) Mean pixel intensity for *TRE-red* measured in discs with physical injury in wild type (88.24 ± 22.58; S.D.) and *p38a* ^*1-/-*^ (70.80 ± 19.14; S.D.). P = 0.33 n.s.

To assess the relationship between JNK and p38, we tested p38 activation in wounded null hemizygous JNKK *hemipterous* (*hep* ^*r75*^) discs. P-p38 was localized near the wound after physical injury (Fig 6A). This contrasts with the decrease in P-p38 when the MAPK kinase *lic*, which is the p38 activating kinase, was interfered with RNAi in injured discs (S6A Fig).

Moreover, P-p38 staining was localized in *hep* ^*r75*^ discs after *ptc>rpr* induction, as in the wild type (Fig 6B, compare with Fig 4), indicating that JNK and p38 act independently. In addition, we fed animals with the JNK Inhibitor IX, which abolishes *TRE-red* reporter expression and inhibits regeneration (S3C and S3D Fig), and found that P-p38 after *rpr*-ablation was not affected (S6B Fig).

To confirm that JNK and p38 act independently, we blocked the *p38* pathway and checked for *TRE-red* reporter activity. As the *p38a* ^*1*^ allele in heterozygosis strongly affects regeneration (Fig 5), we used this null allele in homozygosis and tested *TRE-red* activity after physical injury. Our results showed that *TRE-red* is induced at the wound edges of *p38a* ^*1-/-*^ mutant discs (Fig 6C and 6D). Together, these results demonstrate that p38 and JNK stress responses act independently in damaged imaginal discs.

### *Upd* expression is triggered by ROS

**Fig 7:**
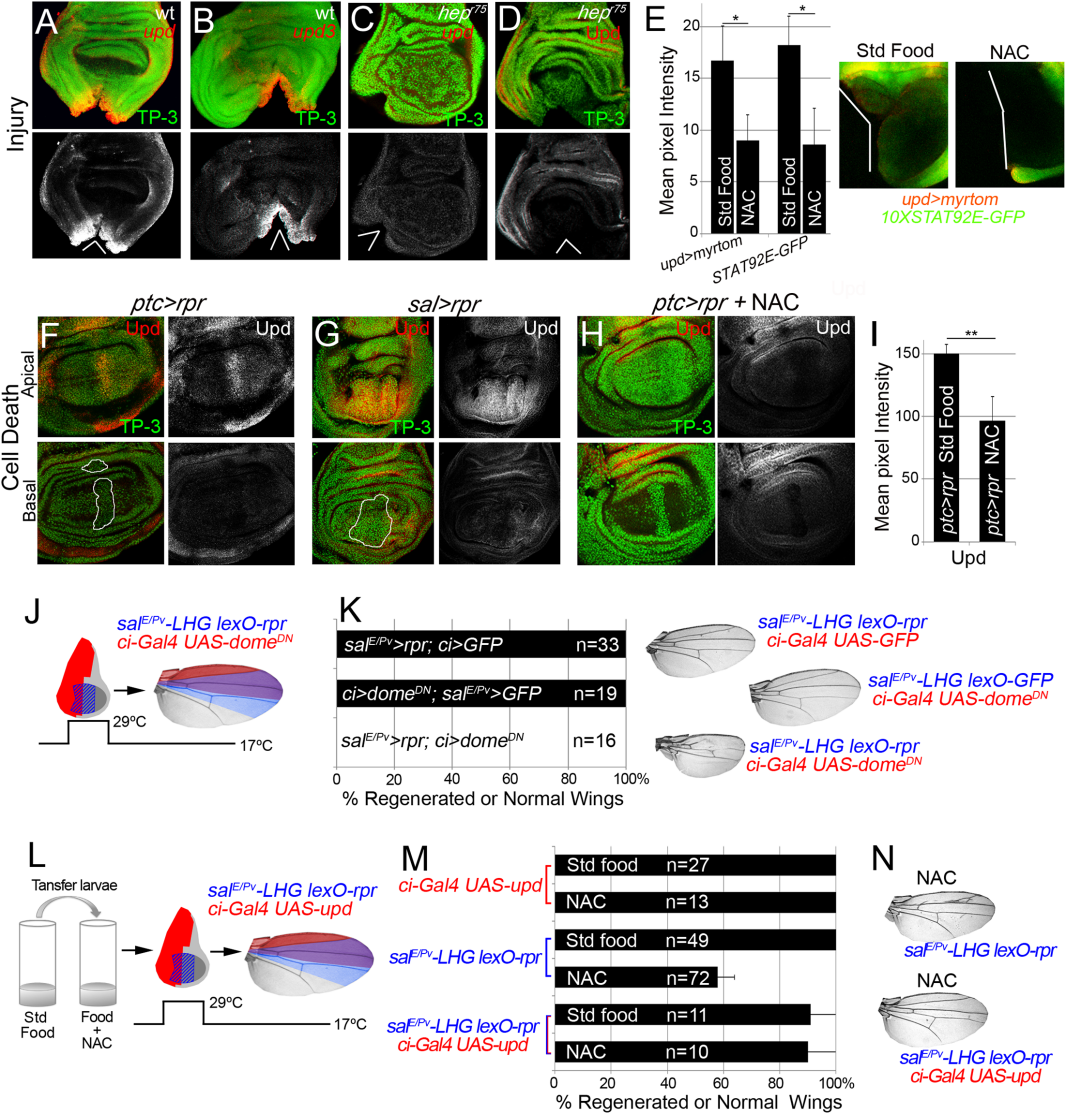
Cytokine signaling is controlled by ROS and JNK. (A, B) In situ hybridization of *upd* (A) and *upd3* (B) in wild type (wt) discs and JNKK *hep* ^*r75*^ hemizygotes (C) after injury. (D) *hep* ^*r75*^ hemizygote stained with anti-Upd after injury. (E) Mean pixel intensities of *upd* reporter (*upd>myrtom* Std food: 16.68 ± 3.22 and NAC: 9.01 ± 3.41, S.D.) and STAT92E reporter (*10xSTAT92E-GFP* Std food: 18.24 ± 2.73 and NAC: 8.63 ± 4.07, S.D.) after standard or NAC-supplemented food. White wedges indicate zone of injury. (F, G) Upd (anti-Upd) is mainly expressed in living cells and not in dead cells after *ptc>rpr* or *sal>rpr*. (H) Upd expression declines after NAC intake. TP-3: TP-PRO-3 nuclei staining. (I) Mean pixel intensities of Upd stained *ptc>rpr* discs with or without NAC feeding (150.29 ± 7.11 and 96.69 ± 18.97, S.D.). (J) Inhibition of the JAK/STAT signaling within *dome* ^*DN*^ impairs wing regeneration. Genetic design (J) using double transactivator system (as in Fig 2) to induce death (blue) and activate *dome* ^*DN*^ (red). (K) Percentage of regenerated wings for controls (*rpr* or *dome* ^*DN*^ expression only) and experimental (*rpr* and *dome* ^*DN*^ dual expression). Note that *dome* ^*DN*^ wings were not able to regenerate (*rpr* and *dome* ^*DN*^ dual expression), whereas *dome* ^*DN*^ wings in the absence of cell death are normal. Examples of wings (left) of controls and experimental. (L) Experimental design for testing the rescue of NAC effects by ectopic activation of *upd*. (M) NAC effect on repair ability was rescued by *upd* overexpression. Quantification of the percentage of wings that regenerate after NAC feeding for the indicated genotypes. (N) Examples of wings from NAC-feeding with *rpr*-ablation defects (upper) and with rescue after *rpr*-ablation and *upd* activation (lower). *P<0.05 **P<0.01.

The evidence that JNK is active in the living tissue located near damaged zones arises from the expression of *puc* and *TRE-red* reporters (Fig 3A and 3B), and also because inhibition of JNK results in defects in repair [21,23,25,26,51]. Moreover, JNK activation promotes *upd* expression in different contexts [28,52]. We wondered whether those low non-deleterious JNK levels are capable of triggering tissue repair through *upd* expression. Upd cytokines are ligands that associate with the receptor *domeless* (*dome*) to stimulate the kinase activity of the receptor associated protein kinase *hopscotch* (*hop*), which in turn phosphorylates dimers of the transcription factor STAT92E [53]. We found *upd* and *upd3* expression near the wound after both physical injury and cell death (Fig 7A, 7B, 7F and 7G). This injury-induced *upd* expression was blocked in JNKK *hep* ^*r75*^ mutants (Fig 7C and 7D) and by JNK Inhibitor IX (S3C Fig), which is consistent with previous observations [28,35].

To study the requirement for JAK/STAT for regeneration, we used the *sal* ^*E/Pv*^ *-LHG lexO-rpr* to induce apoptosis and simultaneously interfered with the receptor *dome* using the dominant negative form *UAS-dome* ^*DN*^ driven by *ci-Gal4*. These wings lacked most of the tissue where cell death was induced and *dome* was blocked (Fig 7J and 7K), indicating that JAK/STAT signaling is needed for tissue recovery. Moreover, heterozygous alleles for the JAK/STAT pathway resulted in partial disruption of adult wing recovery after cell death (S7 Fig).

We next analyzed if JAK/STAT signaling requires ROS in this context. Two different reporters (*10XSTAT92E-GFP* and *upd-Gal4 UAS-myrtomato*) were used in physically injured discs and showed reduced expression after NAC feeding (Fig 7E). In addition, *ptc>rpr* induced discs from NAC fed larvae showed a reduction of *upd* expression (Fig 7H and 7I). Thus, this expression is ROS dependent after both physical injury and cell death. We speculated that if ROS operate upstream *upd*, the impairment of regeneration resulting from NAC feeding should be rescued by activating Upd. To this aim, we used the double transactivation system to induce cell death in NAC-fed individuals and concomitantly activate *upd* expression (Fig 7L). Analysis of the resulting wings showed that *upd* ectopic expression rescued the NAC inhibition phenotype (Fig 7M and 7N). These observations demonstrate that ROS function upstream of JAK/STAT during repair.

**Fig 8:**
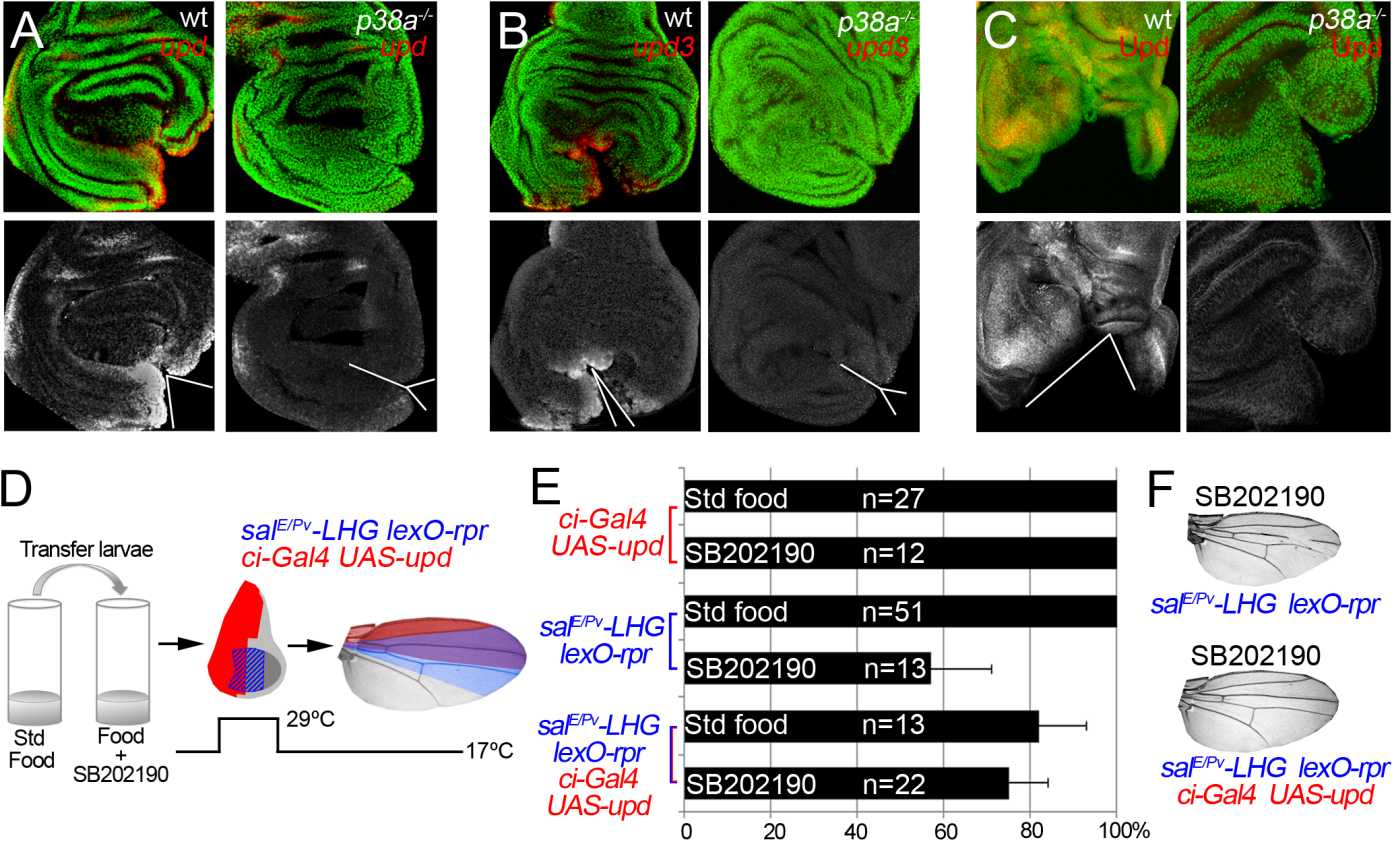
p38 controls *upd* expression. (A) In situ hybridization of *upd* in wild type (wt) and *p38a* ^*1-/-*^ cut discs. (B) In situ hybridization of *upd3* in wild type (wt) and *p38a* ^*1-/-*^ cut discs. (C) Immunostaining with anti-Upd in wild type (wt) and *p38a* ^*1-/-*^ cut discs. White lines and wedges indicate the position of the cut (D) Experimental design for testing the rescue of SB202190 effects by ectopic activation of *upd*. (E) SB202190 effect on repair ability was rescued by *upd* overexpression. Quantification of the percentage of wings that regenerate after SB202190 feeding for the indicated genotypes. (F) Examples of wings from SB202190-feeding with *rpr*-ablation defects (upper) and with rescue after *rpr*-ablation and *upd* activation (lower).

We wondered whether p38 is also required for *upd* expression in damaged discs. Expression of *upd* or *upd3* was severely reduced in *p38a* ^*1-/-*^ wound edges (Figs 8A–8C and S8). This suggests that in addition to JNK, p38 is essential for *upd* expression upon stress. Finally, we argued that if p38 is required for repair through *upd*, its ectopic expression should rescue the impaired regeneration after inhibition of p38. We, again, used the double transactivation system to induce cell death in SB202190-fed individuals, to block p38 phosphorylation and alongside activate *upd* expression (Fig 8D). Indeed, we found that the number of wings that regenerated after p38 inhibition increased (Fig 8E and 8F). Altogether these results position Upd cytokines downstream from the ROS/p38/JNK module.

## Discussion

In this work, we demonstrate a stress-responsive module activated upon cell death or physical damage. This module consists of ROS dependent stimulation of non-deleterious levels of JNK and p38 MAP kinases necessary for the expression of Upd and JAK/STAT signaling which drives regeneration. Non-lethal levels of JNK may have multiple functions, among them cytoskeleton organization [44,54], healing and initiation of regenerative growth [21,23–26,51,55,56]. Thus, this early responsive module is crucial to maintain tissue in a healthy condition, trigger tissue repair and restore homeostasis.

In an apoptotic context, Rpr dimerizes and, through direct binding, brings the *Drosophila* inhibitor of apoptosis protein-1 (DIAP1) to mitochondria, concomitantly promoting DIAP1 auto-ubiquitination and destruction [57,58]. Rpr action on the mitochondria results in alteration of cytochrome C driven by caspases [59] and in mitochondrial disruption [60]. The ROS dyes used here detect a wide range of ROS, and therefore we cannot discriminate between membrane oxidases or mitochondrial origin. However, since Rpr acts on mitochondria, mitochondrial alterations could cause the burst of ROS in apoptotic cells. Of note, we observed that high ROS levels are associated with high levels of JNK in apoptotic cells. It has been proposed that ROS can mediate the activation of JNK [61] by quenching the MAP kinase phosphatases [62]. Conversely, low levels of ROS detected in nearby surviving tissue correlate with low non-deleterious levels of JNK and activation of MAP kinase phosphatases. Thus, *puc* MAP kinase phosphatase could protect the living cells close to the damage from the noxious effects of high JNK. Indeed, living cells near the wound retain low levels of JNK, not sufficient to kill but necessary for tissue recovery.

Additionally, the caspase Dronc, which acts downstream from Rpr, has functions beyond apoptosis [63]. Dronc is involved in the activation of JNK and p53, which activate the pro-apoptotic genes, creating an amplification loop that ensures apoptosis [27,29–31,64]. The JNK/p53 driven apoptosis stimulates proliferation of the nearby tissues [29–31,65]. Although still unclear, it has been proposed that apoptotic cells can release the products of mitogenic genes such as *wingless* (*wg*) and *decapentaplegic* (*dpp*) [33,66,67][31,68]. Alternatively, we show here that ROS operate as signals responding to insults (apoptosis, mechanical stress) that turn on the homeostatic machinery to compensate the epithelial damage. This fits with a scenario in which ROS are able to either diffuse from cell to cell or perhaps to propagate their production to several rows of cells. Indeed, ROS have been proven to cross cell membranes, to spread through gap junctions [69–71] and to enter into the cell through specific membrane aquaporin channels [71,72]. Therefore, ROS behave as an efficient paracrine signal that ultimately will result in Upd activation.

In addition to JNK, ROS are stressors involved in p38 activation [73]. ROS may activate the p38 pathway through the oxidative modification of intracellular kinases such as redox-sensitive activating protein-1 ASK1 [74]. We showed here that not only JNK but also p38 is required for regeneration. Moreover, the *p38a* ^*1*^ allele seems to particularly affect *upd* expression and regeneration. This concurs with the finding that *Drosophila* p38a is more susceptible to environmental stressors, such as oxidative stress [18]. However, other p38 kinases could contribute to tissue regeneration. Indeed, heterozygous alleles of the p38 activating kinase *lic*, which normally do not show patterning defects after *rpr*-mediated ablation, can result in incomplete regeneration when a dose of *p38b* is missing (Fig 5A). Moreover, RNAi of *p38b* also can show defective regeneration individuals (S2 Fig). In addition, we cannot discard that *p38c*, which has been recently found involved in intestinal immune homeostasis [75], may also function in imaginal disc regeneration.

**Fig 9:**
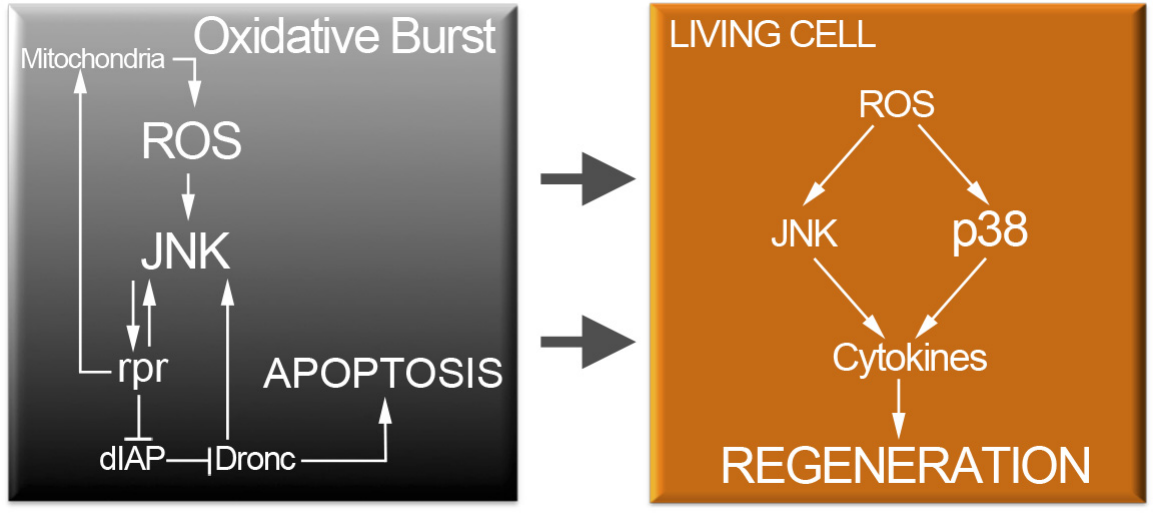
Cell protection module activated by injury or cell death. Oxidative stress in dying cells is likely of mitochondrial origin and results in highly toxic JNK. However, low levels of ROS propagate to adjacent surviving cells (arrows). Non-deleterious ROS will activate moderate levels of JNK and p38 only in surviving cells. P38 and JNK are required for cytokine activation and tissue repair.

We have found that both *hep* ^*r75*^ and *p38a* ^*1*^ inhibit *upd* expression. But *hep* ^*r75*^ mutants, which block JNK signaling, do not affect p38 phosphorylation and viceversa, *p38a* ^*1*^ mutants, which block at least the p38a branch of the p38 kinase, do not interfere with the *TRE-red* reporter expression. This suggests that ROS activate p38 and JNK independently and that both MAP kinases act on *upd* expression to drive tissue repair. Thus, ROS signaling operates through these two MAP kinase pathways that in turn will converge to stimulate the transcriptional expression of the cytokines (Fig 9).

JNK and p38 are not only activated after cell death but also after physical injury. Beneficial ROS production is an ubiquitous reaction associated with inflammatory responses to wounding [4,6,76]. Recent findings show that ROS produced in dynamic epithelia operate as a tuning mechanism for reorganization of epithelia [77]. Therefore, it could be that changes in mechanical stress generated during wounding and epithelial disruption (mechanical stretching) results in ROS production. Some dead cells were also found after physical injury. Thus, a partial contribution of dead cells in addition to the stress due to epithelial disruption can account for the oxidative burst generated after physical injury.

In summary, an early boost of oxidative stress is required to activate p38 and JNK in apoptotic cells or near the wound. Moreover, *upd* is turned on downstream JNK and p38. Thus, downstream of the stress response module, cytokines operate to control tissue growth during regeneration.

## Materials and Methods

### *Drosophila* strains

The *Drosophila melanogaster* strains used were *ptc-Gal4* [78], *tubGal80* ^*TS*^ [79], *UAS-rpr* [80], *ci-Gal4* [81], *nub-Gal4* [82] *sal-Gal4* and *sal* ^*E/Pv*^ *-Gal4* [83], *p38b* ^*d27*^, *lic* ^*d13*^ [47], *dATF2* ^*PB*^ [49], *p38a* ^*1*^, [17], *LexO-rCD2*::*GFP* [43], *TRE-DsRed*.*T4* [45] as *AP1* reporter, *puc-lacZ* [44], *puc* ^*E69-A*^ *-Gal4* [84], *UAS-upd* [85], *upd-Gal4* (from D. Harrison), *10XSTAT92E-GFP* [86], *en-Gal4*, *UAS-GFP*, *UAS-myrtomato*, *UAS-Sod*.*A* (*sod1*), *UAS-Cat*.*A*, *UAS-dome* ^*DN*^, *hop2*, *hop* ^*27*^, *stat92e* ^*06346*^ (Bloomington Stock center), *stat92e* ^*397*^ [87], and *hep* ^*r75*^ [88]. Transgenic *Drosophila shRNAi* lines were obtained from the Vienna Drosophila RNAi Center (VDRC). Canton S was used as the wild type control.

### Imaginal disc culture and physical injury

Wing discs were dissected from third instar larvae in Schneider’s insect medium (Sigma-Aldrich) and a small fragment was removed with tungsten needles. Discs were cultured in Schneider’s insect medium supplemented with 2% heat activated fetal calf serum, 2.5% fly extract and 5 μg/ml insulin, for different periods of time (from 1 to 10 hours) at 25°C. Ex vivo images were taken using a Leica SPE confocal microscope and processed with Fiji software.

### Generation of LexA/lexO strains for genetic ablation

The *sal* ^*E/Pv*^ *-LHG* construct was created cutting the wing specific enhancer of *spalt*, *sal* ^*E/Pv*^ [83] from *pC4LacZ-Spalt PE* EcoRI/BamHI and cloning this fragment into the plasmid *attB-LHG* containing a *Gal80*-suppressible form of *LexA* transcriptional activator (*LHG*) [43]. *LHG* contains both the binding domain of *LexA* and the activator domain of *Gal4*, which is recognized by the inhibitor *Gal80* ^*TS*^.The *LexO-rpr* strain was obtained subcloning the pro-apoptotic gene *reaper* (*rpr*) from *pOT2-rpr* (IP02529) EcoRI/XhoI in the *pLOTattB* plasmid [89] carrying the *lexA* operator *LexO*. Transgenic flies were performed with standard protocols.

### Genetic ablation and dual Gal4/LexA transactivator system

Cell death was genetically induced as previously described [23,90]. We used two different drivers to induce cell death. The first, *ptc-Gal4* which is expressed in a narrow stripe in the center of the disc. This strain was used to induce cell death in imaginal discs (*UAS-rpr*), because the dead domain can be easily discerned from the neighboring living domain. The second, *sal* ^*E/Pv*^ *-Gal4* strain, which consists of *sal* wing enhancer with expression confined to the wing [83] has been used in this work to score adult wing parameters.

The UAS line used to promote cell death was *UAS-rpr*, and the system was controlled by the thermo sensitive repressor *tubGal80* ^*TS*^. We also used the *sal* ^*E/Pv*^ *-LHG* and *LexO-rpr* strains for genetic ablation using the same design as for *Gal4/UAS*.

Embryos were kept at 17°C until the 8th day/192 h after egg laying (equivalent to 96 hours at 25°C) to prevent *rpr* expression. They were subsequently moved to 29°C for 11 hours and then back to 17°C until adulthood. Controls without *rpr* expression were always treated in parallel.

In dual transactivation experiments, we used the *sal* ^*E/Pv*^ *-LHG LexO-rpr* to ablate the *sal* ^*E/Pv*^ domain, whereas *Gal4* was used to express different transgenes under the control of *nub-Gal4* or *ci-Gal4*.

In the experiments on antioxidants (Fig 2) and *upd* (Figs 7 and 8) overexpression, larvae were transferred to NAC- (100 μg/ml) or SB202190- (5 μM) supplemented food 24 h before cell death induction.

### ROS detection ex vivo

All experiments for ROS detection were done in living conditions. To detect the presence of ROS we used CellROX Green Reagent (Life Technologies), which is an indicator of oxidative stress in living cells. For both genetic ablation and physical injury experiments, third instar discs were dissected in Schneider´s medium immediately after cell death or injury and incubated for 15 minutes in medium containing 5 μM CellROX Green Reagent, followed by three washes. Samples were protected from light throughout. Then they were mounted using culture medium supplemented with 1 μM TO-PRO-3 (Life Technologies) nucleic acid stain. As TO-PRO-3 only enters dead cells, we used it to distinguish dead cells from living cells in the ex vivo experiments. Images were taken using a Leica SPE and SPII confocal microscope. Grey values of regions of interest (ROI) were measured using Fiji software. ROIs were established at the wound edges of injured discs (examples in S2 Fig), or in rectangles as indicated in Fig 1E. Pixel intensities were collected and analyzed from raw images taken under the same laser confocal conditions. Thermal LUT images were rendered from slices taken from the confocal using the Interactive 3D Surface Plot tool of the Fiji software (ImageJ). We also used the cell-permeant 2',7'-dichlorodihydrofluorescein diacetate (H2DCFDA 5μM, Life Technologies) which upon oxidation is converted to the highly fluorescent 2',7'-dichlorofluorescein (DCF).

To visualize the ROS images in Fig 1 after genetic ablation, the whole stacks were subject to the Enhance Contrast tool at 0.4 pixel saturation in whole stack normalization. For physical injury images, thermal LUT images were obtained from raw stacks.

### ROS scavenging

To prevent ROS production, we used two protocols. The first was mainly used for *rpr*-ablation discs. It consisted in that antioxidants were supplemented into standard fly food. As antioxidants we used vitamin C (250 μg/ml), Trolox (an analog of vitamin E; 20 μg/ml) and N-acetyl cysteine (NAC) (100 μg/ml), all from Sigma-Aldrich. To score adult wings, larvae were transferred from vials containing standard food to vials containing food with the desired antioxidant concentration. Antioxidant treatment was administered at 168 h of development at 17°C (equivalent to 84 h AEL at 25°C). After 24 hours, experimental larvae were moved to 29°C for 11 hours to promote *rpr* apoptosis. Meanwhile one control consisted of larvae maintained at 17°C and another control consisted of larvae transferred to a vial with standard food and moved to 29°C for the same period as in the experimental group. After *rpr* induction temperature was returned to 17°C to allow tissue recovery. This protocol was applied for Figs 2A–2D, 3D, 3E, 4E, 4F and 7E.

The second was used for ex vivo cultured discs. Wing imaginal discs were incubated for 30 minutes in Schneider’s insect medium supplemented with NAC 100 μg/ml. Then, they were transferred to Schneider’s containing CellROX Green (S2 Fig). NAC incubated discs were used for monitoring *TRE-red* (Fig 3F and 3G) or for P-p38 antibody staining (Fig 4B and 4C). In S2 Fig medium was supplemented with NAC, Trolox or Vit C.

### Chemical inhibition of p38 and JNK pathway

The imidazole drug SB202190 (Sigma-Aldrich) was added to standard fly food to prevent p38 activation. We used three different concentrations (0.12 μM, 1 μM and 5 μM), and DMSO as the control. To inhibit chemically JNK we used the JNK Inhibitor IX (5 μM, Selleckchem) which is a thienylnaphthamide compound that is a selective and potent inhibitor of the ATP binding site of JNK. The timing and protocol followed to inhibit both pathways was the same as that to scavenge ROS.

### Oxidative stress induction

Third instar larvae were transferred to vials containing 1% H_2_O_2_, 1,3% low melting agarose and 5% sucrose. To avoid loss of oxidative capacity, H_2_O_2_ was added at a temperature under 45°C. Larvae were fed for 2h in this medium prior dissection and fixation of the discs. Controls without H_2_O_2_ were done in parallel.

### Test for regenerated adult wings and statistics

For testing the capacity to regenerate we used adult wings emerged from *sal* ^*E/Pv*^ *>rpr* individuals, in which patterning defects can be easily scored. Flies were fixed in glycerol:ethanol (1:2) for 24 h. Wings were dissected on water and then washed with ethanol. Then they were mounted on 6:5 lactic acid:ethanol and analyzed and imaged under a microscope.

Definition of regenerated/non-regenerated wings: when veins or interveins were missing, we considered them as defective in their capacity to restore the normal pattern. Therefore, the % of regenerated wings (Figs 2, 5, 7, 8, S2, S3, S5 and S7) was calculated after the number of wings with the complete set of veins and interveins. For each sample of “regenerated wings” we scored the percentage of individuals that belong to the “regenerated wings” class and calculated the standard error of sample proportion based on binomial distribution (regenerate complete wing or not) SE = √p (1-p)/n, where p is the proportion of successes in the population.

Ratios between wing areas (Fig 5) were used as an indication of the size achieved after cell death for each genetic background, and consisted of a comparison between wing size with and without *rpr* induction.

### Immunochemistry and fluorescence in situ hybridization (FISH)

Immunostaining and FISH were performed using standard protocols. Primary antibodies used in this work were P-p38 (rabbit 1:50, Cell Signaling Technology), phospho-Histone-H3 (rabbit 1:1000, Millipore), ß-galactosidase (rabbit 1:1000, ICN Biomedicals), Upd (rabbit 1:800, gift from D. Harrison) and cleaved caspase-3 (rabbit 1:100, Cappel).

Fluorescently labeled secondary antibodies were from Life Technologies and Jackson Immunochemicals. Discs were mounted in SlowFade (Life Technologies) supplemented with 1 μM TO-PRO-3 (Life Technologies) to label nuclei. Note that in fixed tissues all nuclei are TO-PRO-3 labeled, whereas in ex-vivo culture only nuclei of dead or dying cells are TO-PRO-3 labeled.

The number of mitosis after analyzing the stacks of confocal images was calculated using Fiji software (Cell counter plug-in). Mitosis were counted for the entire anterior compartment of the wing pouch for each disc.

For apoptotic cell detection, we used both anti cleaved caspase 3 or TUNEL assay. For TUNEL we used the fluorescently labeled dUTP ChromaTide BODIPY FL-14-dUTP (Life Technologies) and incorporated using terminal deoxynucleotidyl transferase (Roche).

EdU was incorporated using the Click-iT EdU Imaging Kit (Life Technologies). Wing discs were dissected after cell death induction and incubated in Schneider’s insect medium supplemented with 1 mg/ml EdU for 5 minutes. Following EdU incorporation, discs were fixed and immunostained.

Riboprobes for *upd and upd3* were synthesized using cDNA clones from DGRC AT1366 and FI03911.

### Genotypes

Fig 1

**A, B.** Wild type

**D, E, F.** *ptc>rpr: UAS-rpr/+; ptc-Gal4/+; tubGal80^TS^/+*

Fig 2

**A, B, C.** *sal^E/Pv^>rpr: UAS-rpr/+; sal^E/Pv^-Gal4/+; tubGal80^TS^/+*

**D.** *ptc>rpr: UAS-rpr/+; ptc-Gal4:tubGal80^TS^/+*

**E, F, G.** *sal^E/Pv^>rpr nub>GFP: w; nub-Gal4/UAS-GFP; sal^E/Pv^-LHG:tubGal80^TS^/lexO-rpr*

sal ^E/Pv^ >rpr nub>Cat: w; nub-Gal4/UAS-Cat; sal^E/Pv^-LHG:tubGal80^TS^/lexO-rpr

sal ^E/Pv^ >rpr nub>Sod: w; nub-Gal4/UAS-Sod; sal^E/Pv^-LHG:tubGal80^TS^/lexO-rpr

sal ^E/Pv^ >rpr nub>Sod:Cat: w; nub-Gal4/UAS-Sod:Cat; sal^E/Pv^-LHG:tubGal80^TS^/lexO-rpr

Fig 3

**A, B.** *ptc>rpr: UAS-rpr/+; ptc-Gal4:tubGal80^TS^/TRE-DsRed.T4; puc-LacZ/+*

**C.** *ptc>rpr: UAS-rpr/+; ptc-Gal4:tubGal80^TS^/+; puc-LacZ/+*

**D, E**. *ptc>rpr: UAS-rpr/+; ptc-Gal4:tubGal80^TS^/TRE-DsRed.T4*

**F, G.** *w; TRE-DsRed.T4*

Fig 4

**A, B, C.** Wild type.

**D, E, F**. *ptc>rpr: UAS-rpr/+; ptc-Gal4/+; tubGal80^TS^/+*

**G.** *w; ci-Gal4/UAS-Sod:Cat; sal^E/Pv^-LHG:tubGal80^TS^/lexO-rpr*

Fig 5

**A.** Control: *w; +; sal^E/Pv^-LHG:tubGal80^TS^/lexO-rpr* (control for *lexO-rpr* on the third chromosome) and *w; lexO-rpr/+; sal^E/Pv^-LHG:tubGal80^TS^/+* (control for *lexO-rpr* on the second chromosome)

lic ^d13/+^: lic^d13^/+; +; sal^E/Pv^-LHG:tubGal80^TS^/lexO-rpr

p38b ^d27/+^: w; p38b^d27^/+; sal^E/Pv^-LHG:tubGal80^TS^/lexO-rpr

p38a ^1/+^: w; lexO-rpr/+; p38a^1^/sal^EPv^-LHG:tubGal80^TS^

*lic^d13/+^p38b^d27/+^: lic^d13^*/+; *p38b^d27^/+; sal^EPv^-LHG:tubGal80^TS^/lexO-rpr*

*dATF2^PB/+^: w; Atf2^PB^*/+; *sal^E/Pv^-LHG:tubGal80^TS^/lexO-rpr*

dATF2 ^PB-/-^: w; Atf2^PB^/Atf2^PB^; sal^E/Pv^-LHG:tubGal80^TS^/lexO-rpr

lic ^d13/+^ dATF2 ^PB/+^: lic^d13^/+; Atf2^PB^/+; sal^E/Pv^-LHG:tubGal80^TS^/lexO-rpr

p38b ^d27/+^ dATF2 ^PB/+^: w; p38b^d27^/Atf2^PB^; sal^E/Pv^-LHG:tubGal80^TS^/lexO-rpr

dATF2 ^PB/+^ p38a ^1/+^: w; Atf2^PB^/lexO-rpr; p38a^1^/sal^EPv^-LHG:tubGal80^TS^

**B.** *sal^E/Pv^>rpr: UAS-rpr/+; sal^E/Pv^-Gal4/+; tubGal80^TS^/+*

Fig 6

**A.** *hep^r75^: hep^r75^/Y*

**B.** *ptc>rpr hep^r75^: hep^r75^/Y; ptc-Gal4:tubGal80^TS^/+; UAS-rpr/+*

**C.** wt: *w; TRE-DsRed.T4*

p38a ^-/-^: w; TRE-DsRed.T4; p38a^1^/p38a^1^

Fig 7

**A, B.** Wild type.

**C, D.** *hep^r75^: hep^r75^/Y*

**E.** *upd-Gal4/+; UAS-myrtomato/10XSTAT92E-GFP*

**F, G, H, I.** *ptc>rpr: UAS-rpr/+; ptc-Gal4/+; tubGal80^TS^/+*

sal ^E/Pv^ >rpr: UAS-rpr/+; sal-Gal4/+; tubGal80^TS^/+

**J, K.** *sal^E/Pv^>rpr ci>GFP: w; ci-Gal4/lexO-rpr; sal^E/Pv^-LHG:tubGal80^TS^/UAS-GFP*

ci>dome ^DN^ sal ^E/Pv^ >GFP: w; ci-Gal4/lexO-rCD2::GFP; sal^E/Pv^-LHG:tubGal80^TS^/UAS-dome^DN^

sal ^E/Pv^ >rpr ci> dome ^DN^: w; ci-Gal4/lexO-rpr; sal^E/Pv^-LHG:tubGal80^TS^/UAS-dome^DN^

**L, M, N.** *ci-Gal4 UAS-upd: w; ci-Gal4/UAS-upd; sal^E/Pv^-LHG:tubGal80^TS^/lexO-rCD2::GFP*

sal ^E/Pv^ -LHG lexO-rpr: w; ci-Gal4/UAS-GFP; sal^E/Pv^-LHG:tubGal80^TS^/lexO-rpr

ci-Gal4 UAS-upd sal ^E/Pv^ -LHG lexO-rpr: w; ci-Gal4/UAS-upd; sal^E/Pv^-LHG:tubGal80^TS^/lexO-rpr

Fig 8

**A, B, C.** Wild type.

p38a ^-/-^: w; +; p38a^1^/p38a^1^

**D, E, F.** *ci-Gal4 UAS-upd: w; ci-Gal4/UAS-upd; sal^E/Pv^-LHG:tubGal80^TS^/lexO-rCD2::GFP*

sal ^E/Pv^ -LHG lexO-rpr: w; ci-Gal4/UAS-GFP; sal^E/Pv^-LHG:tubGal80^TS^/lexO-rpr

ci-Gal4 UAS-upd sal ^E/Pv^ -LHG lexO-rpr: w; ci-Gal4/UAS-upd; sal^E/Pv^-LHG:tubGal80^TS^/lexO-rpr

S1 Fig

**A, C, D.** Wild type.

**B.** *ptc>rpr: UAS-rpr/+; ptc-Gal4:tubGal80^TS^/+*

S2 Fig

**A.** wt

**B.** *sal^E/Pv^>rpr: UAS-rpr/+; sal^E/Pv^-Gal4/+; tubGal80^TS^/+*

**C.** *sal^E/Pv^>rpr nub>GFP: w; nub-Gal4/UAS-GFP; sal^E/Pv^-LHG:tubGal80^TS^/lexO-rpr*

sal ^E/Pv^ >rpr nub>Cat: w; nub-Gal4/UAS-Cat; sal^E/Pv^-LHG:tubGal80^TS^/lexO-rpr

sal ^E/Pv^ >rpr nub>Sod: w; nub-Gal4/UAS-Sod; sal^E/Pv^-LHG:tubGal80^TS^/lexO-rpr

sal ^E/Pv^ >rpr nub>Sod:Cat: w; nub-Gal4/UAS-Sod:Cat; sal^E/Pv^-LHG:tubGal80^TS^/lexO-rpr

**D.** *sal^E/Pv^>rpr nub>GFP: w; nub-Gal4/UAS-GFP; sal^E/Pv^-LHG:tubGal80^TS^/lexO-rpr*

sal ^E/Pv^ >rpr nub>Cat: w; nub-Gal4/UAS-Cat; sal^E/Pv^-LHG:tubGal80^TS^/lexO-rpr

nub>Cat: w; nub-Gal4/UAS-Cat; sal^E/Pv^-LHG:tubGal80^TS^/lexO-GFP

sal ^E/Pv^ >rpr nub>Sod: w; nub-Gal4/UAS-Sod; sal^E/Pv^-LHG:tubGal80^TS^/lexO-rpr

nub>Sod: w; nub-Gal4/UAS-Sod; sal^E/Pv^-LHG:tubGal80^TS^/lexO-GFP

sal ^E/Pv^ >rpr nub>Sod:Cat: w; nub-Gal4/UAS-Sod:Cat; sal^E/Pv^-LHG:tubGal80^TS^/lexO-rpr

nub>Sod:Cat: w; nub-Gal4/UAS-Sod:Cat; sal^E/Pv^-LHG:tubGal80^TS^/lexO-GFP

S3 Fig

**A.** Wild type.

**B.** *w; TRE-DsRed.T4/+; puc-Gal4:UAS-GFP/+*

**C.** *ptc>rpr: UAS-rpr/+; ptc-Gal4:tubGal80^TS^/TRE-DsRed.T4*

**D.** *sal^E/Pv^>rpr: UAS-rpr/+; sal^E/Pv^-Gal4/+; tubGal80^TS^/+*

S4 Fig

Wild type

S5 Fig

**A.** *sal^E/Pv^>rpr: w; ci-Gal4/UAS-GFP; sal^E/Pv^-LHG:tubGal80^TS^/lexO-rpr*

ci>RNAi p38a: w; ci-Gal4/UAS-RNAi p38a; sal^E/Pv^-LHG:tubGal80^TS^/lexO-GFP

sal ^E/Pv^ >rpr ci>RNAi p38a: w; ci-Gal4/UAS-RNAi p38a; sal^E/Pv^-LHG:tubGal80^TS^/lexO-rpr

ci>RNAi p38b: w; ci-Gal4/UAS-RNAi p38b; sal^E/Pv^-LHG:tubGal80^TS^/lexO-GFP

sal ^E/Pv^ >rpr ci>RNAi p38b: w; ci-Gal4/UAS-RNAi p38b; sal^E/Pv^-LHG:tubGal80^TS^/lexO-rpr

ci>RNAi Atf2: w; ci-Gal4/UAS-RNAi Atf2; sal^E/Pv^-LHG:tubGal80^TS^/lexO-GFP

sal ^E/Pv^ >rpr ci>RNAi Atf2: w; ci-Gal4/UAS-RNAi Atf2; sal^E/Pv^-LHG:tubGal80^TS^/lexO-rpr

ci>RNAi lic: w; ci-Gal4/UAS-RNAi lic; sal^E/Pv^-LHG:tubGal80^TS^/lexO-GFP

sal ^E/Pv^ >rpr ci>RNAi lic: w; ci-Gal4/UAS-RNAi lic; sal^E/Pv^-LHG:tubGal80^TS^/lexO-rpr

**B.** *sal^E/Pv^>rpr: UAS-rpr/+; sal^E/Pv^-Gal4/+; tubGal80^TS^/+*

**C.** *sal^E/Pv^>rpr: UAS-rpr/+; sal^E/Pv^-Gal4/+; tubGal80^TS^/+*

ptc>rpr: UAS-rpr/+; ptc-Gal4:tubGal80^TS^/TRE-DsRed.T4

S6 Fig

en>RNAi lic: w; en-Gal4:UAS-GFP/UAS-RNAi lic

S7 Fig

Control: *UAS-rpr/+; sal^E/Pv^-Gal4/+; tubGal80^TS^/+*

hop ^2/+^: UAS-rpr/hop^2^; sal^E/Pv^-Gal4/+; tubGal80^TS^/+

hop ^27/+^: UAS-rpr/hop^27^; sal^E/Pv^-Gal4/+; tubGal80^TS^/+

stat92e ^397/+^: UAS-rpr/+; sal^E/Pv^-Gal4/+; tubGal80^TS^/stat92e^397^

stat92e ^06346/+^: UAS-rpr/+; sal^E/Pv^-Gal4/+; tubGal80^TS^/stat92e^06346^

S8 Fig

Wild type.

p38a ^-/-^: w; +; p38a^1^/p38a^1^

## Supporting Information

S1 Fig Additional data on ROS activation after damage. (A) Propagation of ROS labeled with CellROX Green towards the adjacent tissue during the first 15’ after injury. Thermal scale corresponds to the same as in Fig 1B. White line indicates cut edge. (B) ROS detected with H2DCFDA after *ptc>rpr*. ROS are found in dead cells and in adjacent living cells. TP-3: TO-PRO-3. (C, D) ROS detected with H2DCFDA after physical injury (white wedge).
(TIF)

S2 Fig Additional controls for Fig 2. (A) Ex vivo analysis of cut imaginal discs cultured in Schneider’s medium, incubated with NAC, Trolox or VitC. Top row shows images of control discs (no antioxidant). Lower row shows images of discs incubated with the indicated antioxidant. Dotted lines indicate zones used as ROI for pixel intensity measurements (below). White wedges indicate the position of the cut. *P<0.05 **P<0.01. (B) Examples of control wings kept at 17°C (*sal* ^*EPv*^ *>rpr OFF*) that grew in food supplemented with antioxidant. All cases, showed normal set of interveins and veins. (C) Examples of control wings kept at 17°C (*sal* ^*EPv*^ *>rpr OFF*) that grew from the indicated genotypes. (D) Controls for transgenes of Fig 2F. Activation of transgenes (*nub>Cat; nub>Sod; nub>Sod*:*Cat*) in the absence of cell death results in normal wings.
(TIF)

S3 Fig Test of JNK reporters and additional data for Fig 3. **(**A) Endogenous expression of the *TRE-red* and *puc-lacZ* reporters. Note that both reporters are expressed only at the tip of the notum (n: notum; wp: wing pouch). (B) *TRE-red* and *puc>GFP* expression after physical injury. Note that *TRE-red* expression is activated earlier and more extensive than *puc>GFP*. White wedges indicate the position of the cut. Dotted line indicates the edges of the disc. (C) The JNK Inhibitor IX eliminates *TRE-red* activity and *upd* expression. Top row: *rpr*–ablated disc from larvae fed with standard food stained for nuclei (TP3: TO-PRO-3), *TRE-red* reporter, and anti-Upd. Bottom row: *rpr*–ablated disc from larvae supplemented with JNK Inhibitor IX. (D) JNK Inhibitor IX inhibits regeneration. Quantification of regenerated *sal* ^*EPv*^ *>rpr* wings after feeding with standard food or JNK Inhibitor IX supplemented.
(TIF)

S4 Fig Additional data for Fig 4. To test whether P-p38 is activated after an independent mechanism of oxidative stress in the absence of damage, larvae were fed with 1% H_2_O_2_ for 2 h before processed for imaging. (A) Live imaging showing high ROS in the entire disc. (B) Fixed disc stained with P-p38.
(TIF)

S5 Fig Additional data for Fig 5. (A) Inhibition of p38 with RNAi constructs prevents tissue repair. Ectopic expression of p38 RNAis under the control of *ci-Gal4* and simultaneous cell death induction with *sal* ^*E/Pv*^ *-LHG LexO-rpr* when shifted to 29°C for 11 h. Adult wing size was measured after ectopic expression of the indicated RNAi transgenes (red). The experiments with *rpr*-ablation are indicated in blue. (B) Examples of control wings of Fig 5B, in which no *rpr*-ablation was induced (kept at 17°C) for the indicated concentrations of the p38 inhibitor SB202190. (C) Test for the reliability of the SB202190. Discs were dissected from *rpr*-ablated larvae that were fed with 5 μM SB202190, fixed and imaged. SB202190 intake reduces P-p38 activation after cell death as measured from the Mean Pixel Intensity in comparison to DMSO fed larvae. *TRE-red* in individuals fed with 5 μM SB202190 is active. Right: Mean Pixel Intensities for both experiments. For p38: control 48.77 ± 30.82 (S.D.); SB202190 10.52 ± 8.09 (S.D.). For *TRE-red*: control 136.46 ± 44.5 (S.D.); SB202190 93.5 ± 23.19 (S.D.). ***P<0.001 for the P-p38 and P = 0,15 n.s. for *TRE-red*.
(TIF)

S6 Fig Additional data for Fig 6. (A) RNA interference of MKK *lic* inhibits p38 phosphorilation after injury. The *UAS-RNAi lic* was activated in the posterior compartment together with *UAS-GFP* (white). Two injuries were inflected with tungsten needles, one in the anterior and one in the posterior compartment. The cuts were performed in Schneider’s medium, and fixation for immunostaining 20’ after injury. P-p38 activation was localized in the anterior compartment and almost absent around the posterior cut. (B) Blocking JNK with JNK Inhibitor IX does not affect P-p38 after *rpr*-ablation. The domain of dead cells is outlined in white.
(TIF)

S7 Fig Loss of JAK/STAT impedes repair after *rpr*-ablation. Percentages of regenerated wings for the indicated genetic background after *sal* ^*E/Pv*^ *>rpr* ablation. Right wings: *sal* ^*E/Pv*^ *>*rpr OFF column: wings of those genetic backgrounds without cell death (kept at 17°C). All wings raised in those conditions contain the normal set of veins and interveins. *sal* ^*E/Pv*^ *>rpr* ON column: top (wt) is an example of fully regenerated wing. The rest of wings are examples of non-regenerated or incomplete regeneration in the heterozygous condition indicated.
(TIF)

S8 Fig Additional data for Fig 8. Quantification of in situ hybridizations of *upd* mRNA (A) and *upd3* mRNA (B) and antibody localization for Upd (C). Regions of interest were determined around the wound edges (as in S2 Fig) of wild type discs (wt) and *p38a* ^*1-/-*^ mutants. Images in Fig 8A–8C are examples of the quantification shown here. ***P<0.001 **P<0.01. Bars indicate standard deviation.
(TIF)
