# Supplementary figures and images for: ROS-Induced JNK and p38 Signaling Is Required for Unpaired Cytokine Activation during Drosophila Regeneration
Source: PLoS Genet. 2015 Oct 23;11(10):e1005595. doi: 10.1371/journal.pgen.1005595 (PMC4619769; doi:10.1371/journal.pgen.1005595)

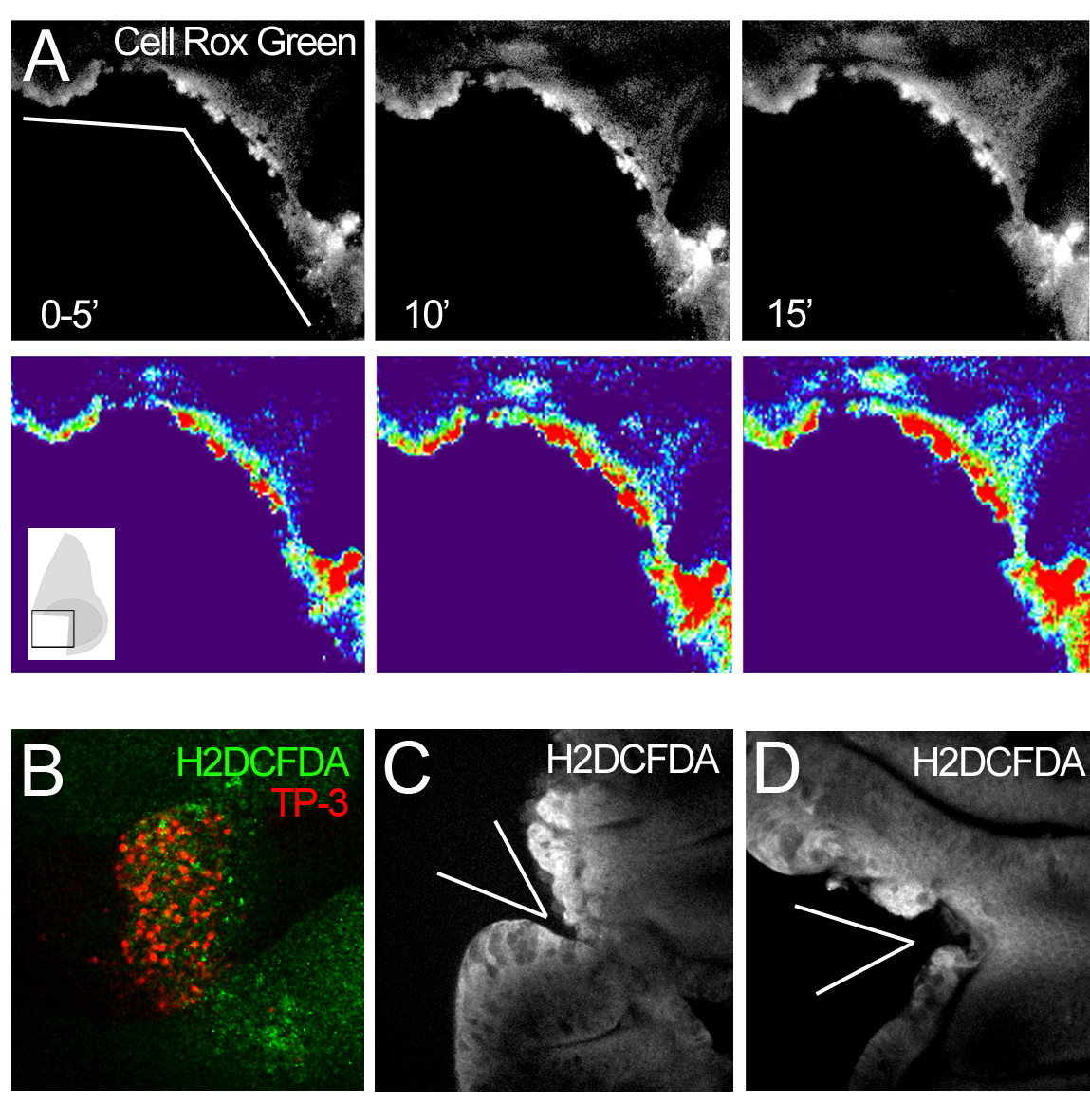

Supplement: S1 Fig — (A) Propagation of ROS labeled with CellROX Green towards the adjacent tissue during the first 15’ after injury. Thermal scale corresponds to the same as in Fig 1B. White line indicates cut edge. (B) ROS detected with H2DCFDA after ptc>rpr. ROS are found in dead cells and in adjacent living cells. TP-3: TO-PRO-3. (C, D) ROS detected with H2DCFDA after physical injury (white wedge). (TIF) [file pgen.1005595.s001.tif]

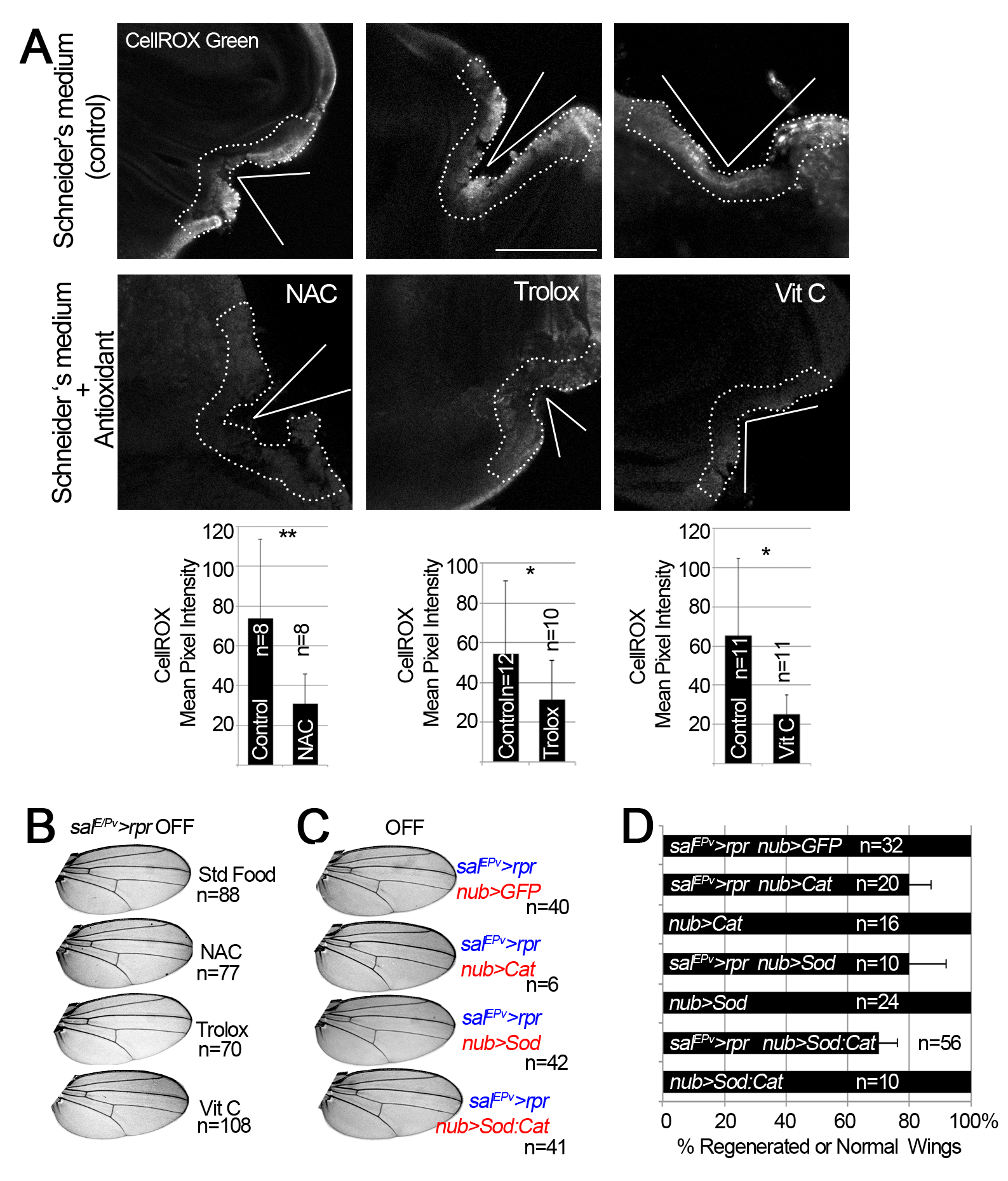

Supplement: S2 Fig — (A) Ex vivo analysis of cut imaginal discs cultured in Schneider’s medium, incubated with NAC, Trolox or VitC. Top row shows images of control discs (no antioxidant). Lower row shows images of discs incubated with the indicated antioxidant. Dotted lines indicate zones used as ROI for pixel intensity measurements (below). White wedges indicate the position of the cut. *P<0.05 **P<0.01. (B) Examples of control wings kept at 17°C (sal EPv >rpr OFF) that grew in food supplemented with antioxidant. All cases, showed normal set of interveins and veins. (C) Examples of control wings kept at 17°C (sal EPv >rpr OFF) that grew from the indicated genotypes. (D) Controls for transgenes of Fig 2F. Activation of transgenes (nub>Cat; nub>Sod; nub>Sod:Cat) in the absence of cell death results in normal wings. (TIF) [file pgen.1005595.s002.tif]

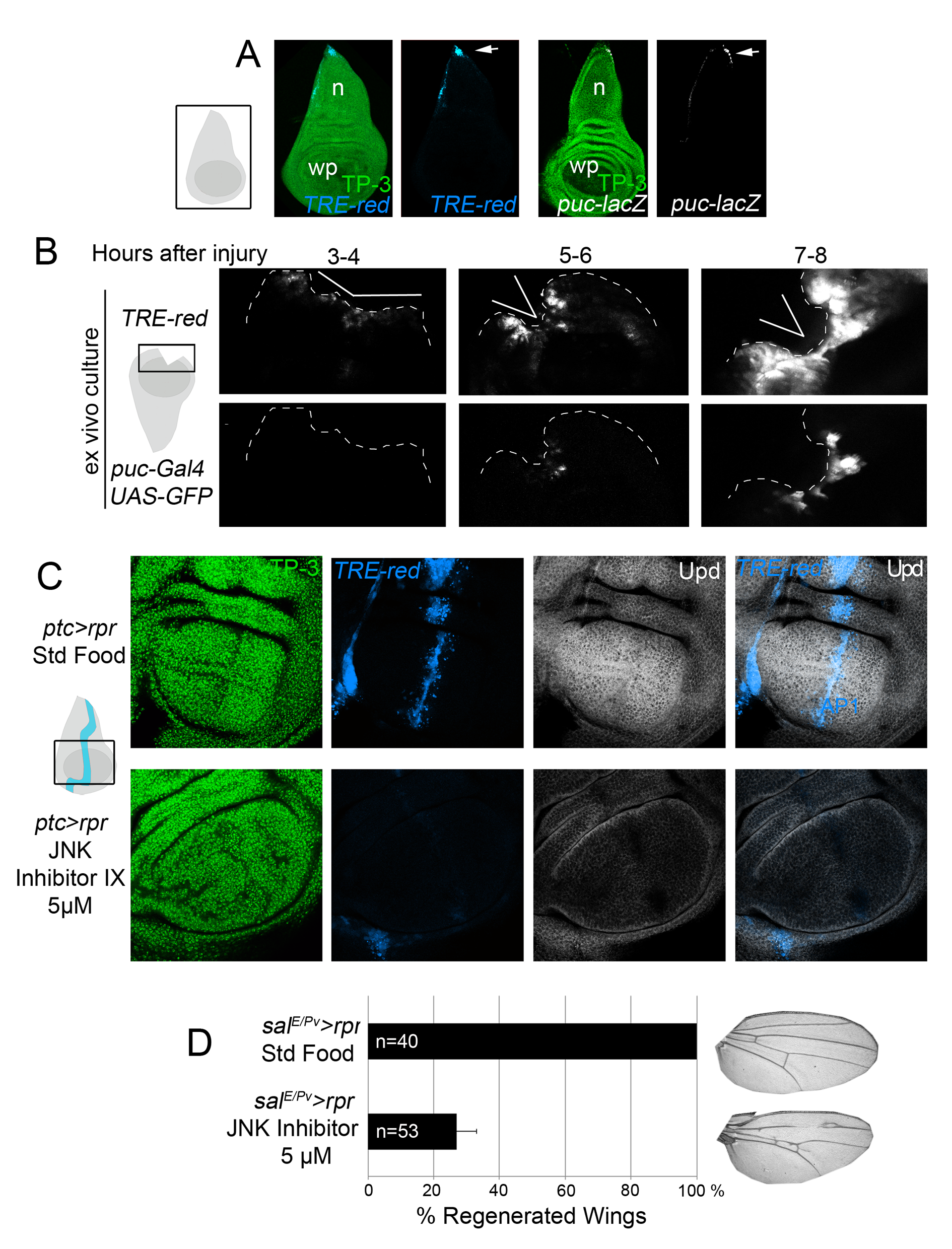

Supplement: S3 Fig — (A) Endogenous expression of the TRE-red and puc-lacZ reporters. Note that both reporters are expressed only at the tip of the notum (n: notum; wp: wing pouch). (B) TRE-red and puc>GFP expression after physical injury. Note that TRE-red expression is activated earlier and more extensive than puc>GFP. White wedges indicate the position of the cut. Dotted line indicates the edges of the disc. (C) The JNK Inhibitor IX eliminates TRE-red activity and upd expression. Top row: rpr–ablated disc from larvae fed with standard food stained for nuclei (TP3: TO-PRO-3), TRE-red reporter, and anti-Upd. Bottom row: rpr–ablated disc from larvae supplemented with JNK Inhibitor IX. (D) JNK Inhibitor IX inhibits regeneration. Quantification of regenerated sal EPv >rpr wings after feeding with standard food or JNK Inhibitor IX supplemented. (TIF) [file pgen.1005595.s003.tif]

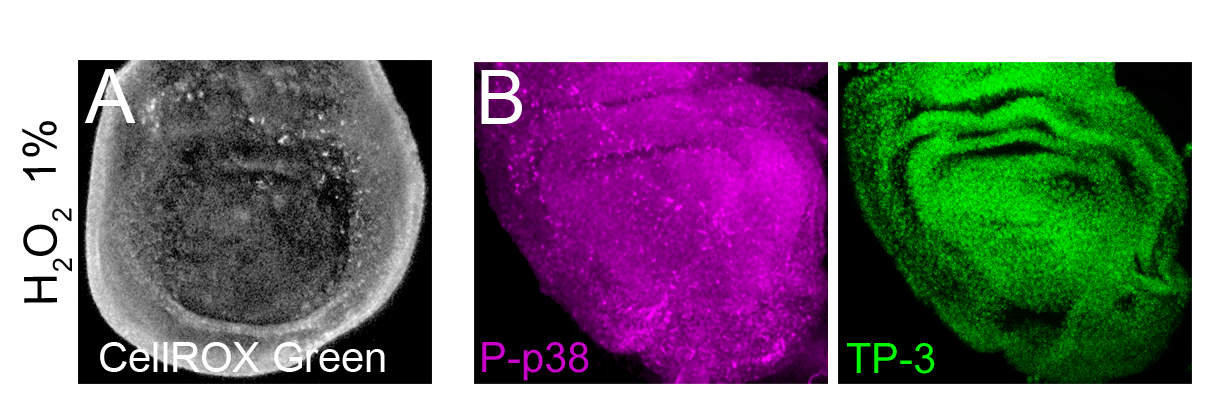

Supplement: S4 Fig — To test whether P-p38 is activated after an independent mechanism of oxidative stress in the absence of damage, larvae were fed with 1% H2O2 for 2 h before processed for imaging. (A) Live imaging showing high ROS in the entire disc. (B) Fixed disc stained with P-p38. (TIF) [file pgen.1005595.s004.tif]

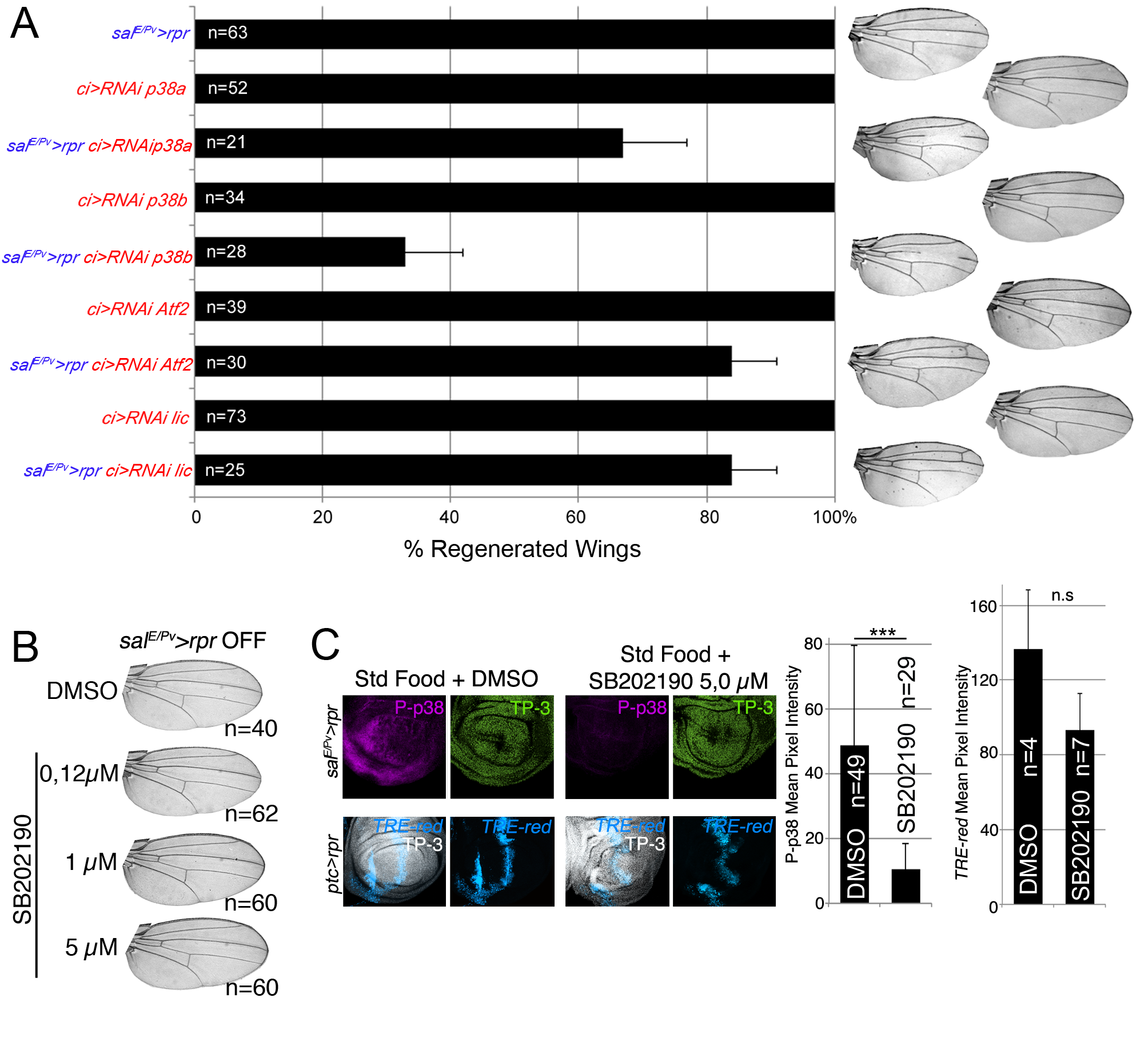

Supplement: S5 Fig — (A) Inhibition of p38 with RNAi constructs prevents tissue repair. Ectopic expression of p38 RNAis under the control of ci-Gal4 and simultaneous cell death induction with sal E/Pv -LHG LexO-rpr when shifted to 29°C for 11 h. Adult wing size was measured after ectopic expression of the indicated RNAi transgenes (red). The experiments with rpr-ablation are indicated in blue. (B) Examples of control wings of Fig 5B, in which no rpr-ablation was induced (kept at 17°C) for the indicated concentrations of the p38 inhibitor SB202190. (C) Test for the reliability of the SB202190. Discs were dissected from rpr-ablated larvae that were fed with 5 μM SB202190, fixed and imaged. SB202190 intake reduces P-p38 activation after cell death as measured from the Mean Pixel Intensity in comparison to DMSO fed larvae. TRE-red in individuals fed with 5 μM SB202190 is active. Right: Mean Pixel Intensities for both experiments. For p38: control 48.77 ± 30.82 (S.D.); SB202190 10.52 ± 8.09 (S.D.). For TRE-red: control 136.46 ± 44.5 (S.D.); SB202190 93.5 ± 23.19 (S.D.). ***P<0.001 for the P-p38 and P = 0,15 n.s. for TRE-red. (TIF) [file pgen.1005595.s005.tif]

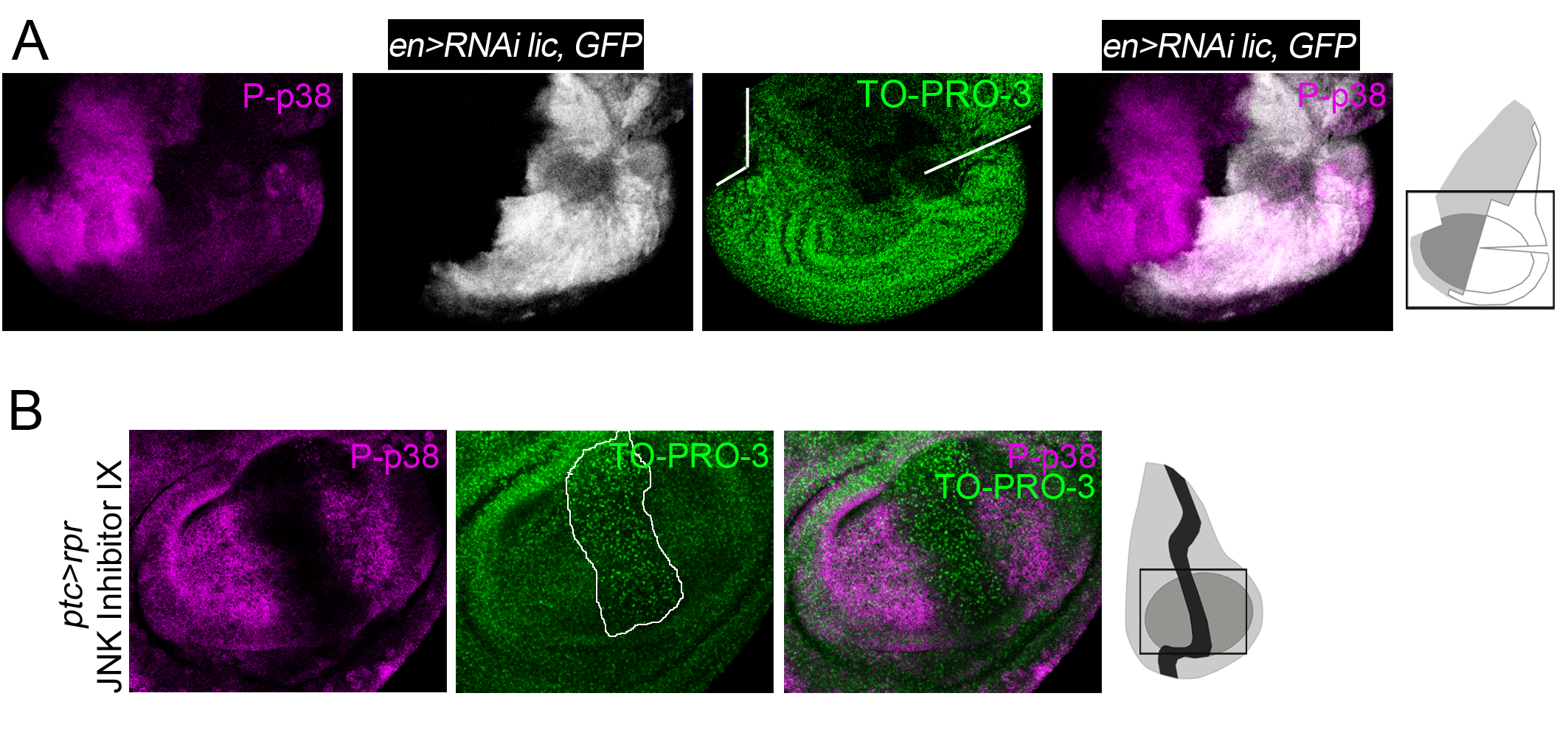

Supplement: S6 Fig — (A) RNA interference of MKK lic inhibits p38 phosphorilation after injury. The UAS-RNAi lic was activated in the posterior compartment together with UAS-GFP (white). Two injuries were inflected with tungsten needles, one in the anterior and one in the posterior compartment. The cuts were performed in Schneider’s medium, and fixation for immunostaining 20’ after injury. P-p38 activation was localized in the anterior compartment and almost absent around the posterior cut. (B) Blocking JNK with JNK Inhibitor IX does not affect P-p38 after rpr-ablation. The domain of dead cells is outlined in white. (TIF) [file pgen.1005595.s006.tif]

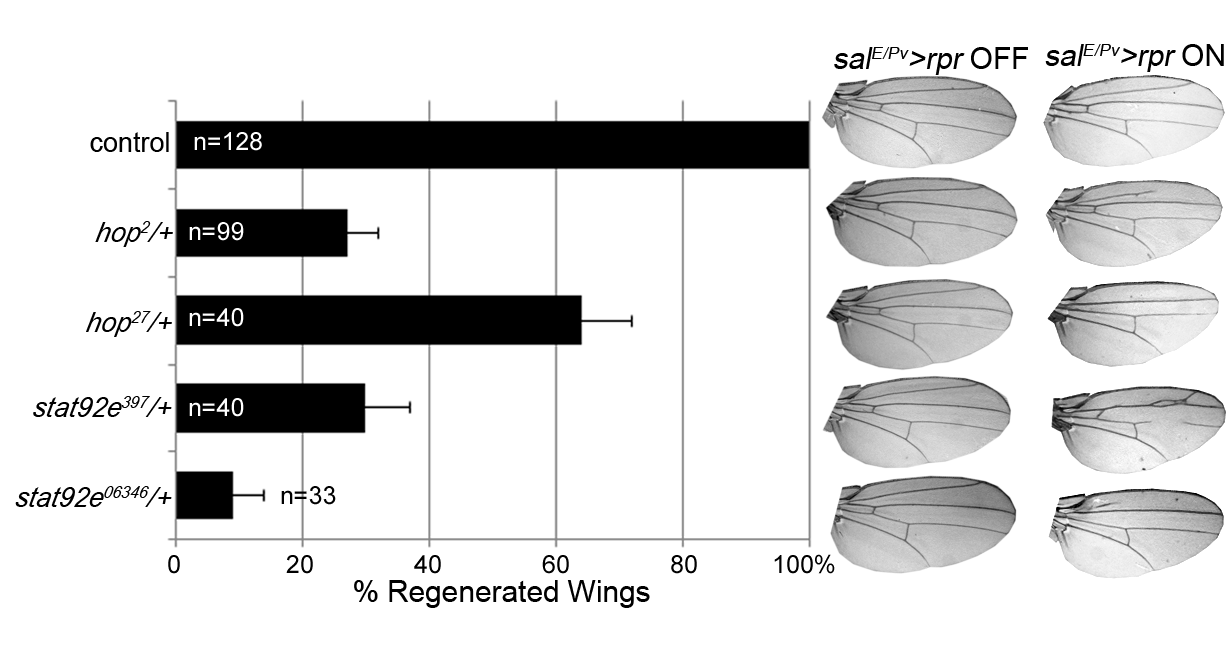

Supplement: S7 Fig — Percentages of regenerated wings for the indicated genetic background after sal E/Pv >rpr ablation. Right wings: sal E/Pv >rpr OFF column: wings of those genetic backgrounds without cell death (kept at 17°C). All wings raised in those conditions contain the normal set of veins and interveins. sal E/Pv >rpr ON column: top (wt) is an example of fully regenerated wing. The rest of wings are examples of non-regenerated or incomplete regeneration in the heterozygous condition indicated. (TIF) [file pgen.1005595.s007.tif]

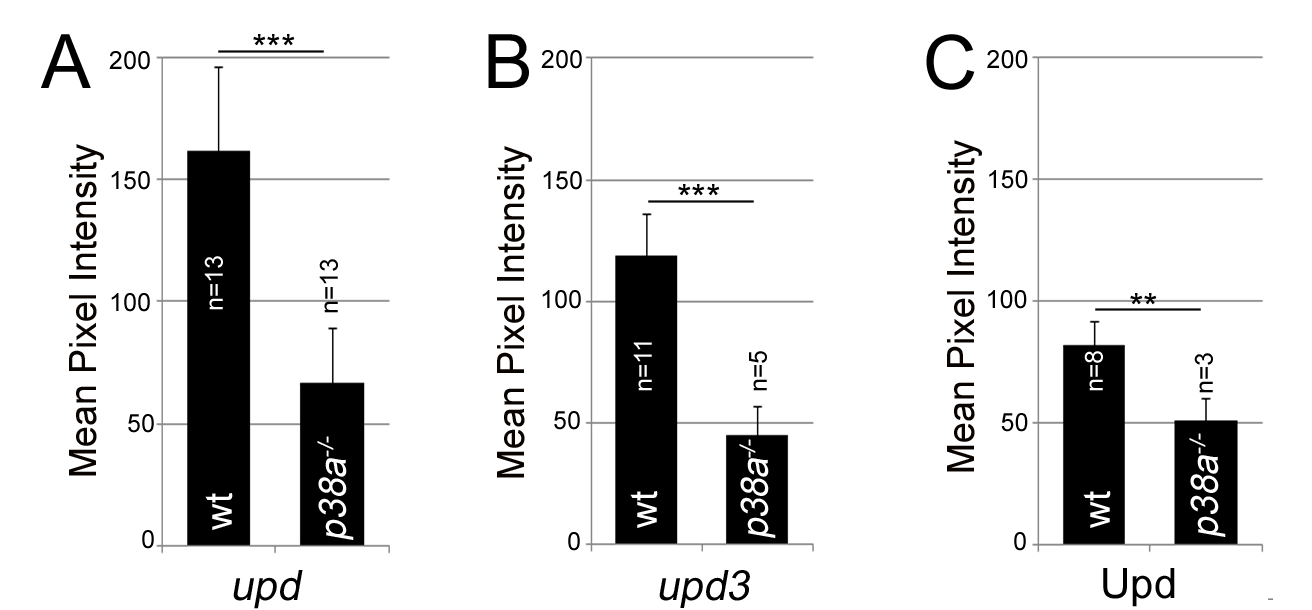

Supplement: S8 Fig — Quantification of in situ hybridizations of upd mRNA (A) and upd3 mRNA (B) and antibody localization for Upd (C). Regions of interest were determined around the wound edges (as in S2 Fig) of wild type discs (wt) and p38a 1-/- mutants. Images in Fig 8A–8C are examples of the quantification shown here. ***P<0.001 **P<0.01. Bars indicate standard deviation. (TIF) [file pgen.1005595.s008.tif]
